# Synergistic antitumor activity by dual blockade of CCR1 and CXCR2 expressed on myeloid cells within the tumor microenvironment

**DOI:** 10.1038/s41416-024-02710-x

**Published:** 2024-05-15

**Authors:** Hideyuki Masui, Kenji Kawada, Yoshiro Itatani, Hideyo Hirai, Yuki Nakanishi, Yoshiyuki Kiyasu, Keita Hanada, Michio Okamoto, Wataru Hirata, Yasuyo Nishikawa, Naoko Sugimoto, Takuya Tamura, Yoshiharu Sakai, Kazutaka Obama

**Affiliations:** 1 Department of Surgery, Graduate School of Medicine, Kyoto University, Kyoto, Japan; 2 Department of Surgery, Kurashiki Central Hospital, Okayama, Japan; 3 Laboratory of Stem Cell Regulation, School of Life Sciences, Tokyo University of Pharmacy and Life Sciences, Tokyo, Japan; 4 Department of Gastroenterology and Hepatology, Kyoto University Graduate School of Medicine, Kyoto, Japan; 5 Rogel Cancer Center, University of Michigan, Ann Arbor, MI USA; 6 Department of Surgery, Rakuwakai Otowa Hospital, Kyoto, Japan; 7 Department of Surgery, Uji-Tokushukai Medical Center, Kyoto, Japan; 8 Department of Surgery, Japanese Red Cross Osaka Hospital, Osaka, Japan

**Keywords:** Cancer microenvironment, Colorectal cancer, Cancer models

## Abstract

**Background:**

Chemokine signaling within the tumor microenvironment can promote tumor progression. Although CCR1 and CXCR2 on myeloid cells could be involved in tumor progression, it remains elusive what effect would be observed if both of those are blocked.

**Methods:**

We employed two syngeneic colorectal cancer mouse models: a transplanted tumor model and a liver metastasis model. We generated double-knockout mice for CCR1 and CXCR2, and performed bone marrow (BM) transfer experiments in which sub-lethally irradiated wild-type mice were reconstituted with BM from either wild-type, *Ccr1*^−/−^, *Cxcr2*^−/−^ or *Ccr1*^−/−^*Cxcr2*^−/−^ mice.

**Results:**

Myeloid cells that express MMP2, MMP9 and VEGF were accumulated around both types of tumors through CCR1- and CXCR2-mediated pathways. Mice reconstituted with *Ccr1*^−/−^*Cxcr2*^−/−^ BM exhibited the strongest suppression of tumor growth and liver metastasis compared with other three groups. Depletion of CCR1^+^CXCR2^+^ myeloid cells led to a higher frequency of CD8^+^ T cells, whereas the numbers of Ly6G^+^ neutrophils, FOXP3^+^ Treg cells and CD31^+^ endothelial cells were significantly decreased. Furthermore, treatment with a neutralizing anti-CCR1 mAb to mice reconstituted with *Cxcr2*^−/−^ BM significantly suppressed tumor growth and liver metastasis.

**Conclusion:**

Dual blockade of CCR1 and CXCR2 pathways in myeloid cells could be an effective therapy against colorectal cancer.

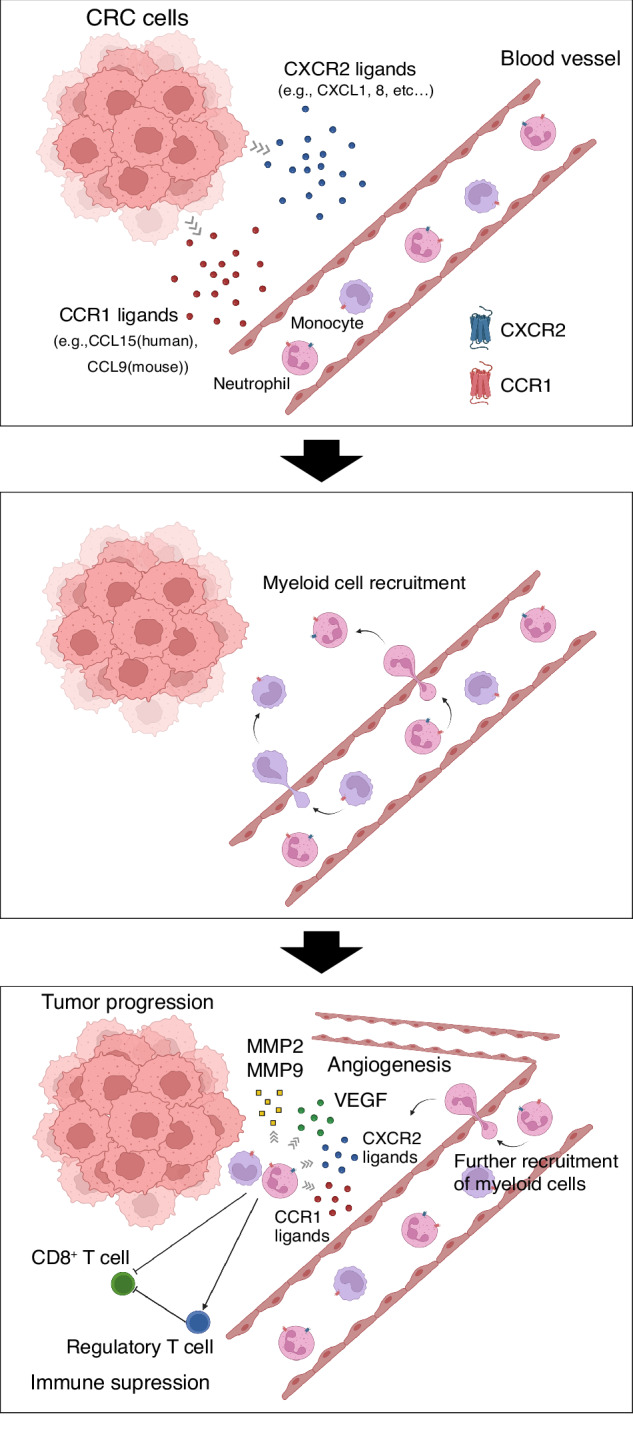

## Background

Colorectal cancer (CRC) is a major global health crisis, characterized by rapidly increasing incidence and mortality rates. Over the past 15 years, mortality from CRC has increased by more than 30%, and this is projected to increase by 25% over the next decade, despite significant advances in surgery, radiotherapy, chemotherapy, and molecular-targeted drugs [1]. Distant metastasis, especially in the liver, is a significant contributor to this dismal prognosis and is common in at least one-third of patients with CRC.

The tumor microenvironment (TME) plays a critical role in tumor progression and metastasis. This dynamic milieu comprises various cellular constituents, including cancer cells, immune cells, stromal cells, and host cells such as macrophages, fibroblasts, and mesenchymal stem cells. Together, they foster rich crosstalk involving signaling pathways such as TGF-β, TNF, and NF-kB, driving tumor growth, invasion, angiogenesis, immune evasion, and metastasis. The interplay between tumor cells and host cells is crucial for cancer progression. Growing interest in cancer research has shed light on the important roles of bone marrow-derived cells (BMDCs) such as neutrophils, monocytes, and myeloid-derived suppressor cells (MDSCs). The involvement of BMDCs in various facets of tumorigenesis - including tumor growth, angiogenesis, epithelial-mesenchymal transition and metastasis – has been increasingly recognized, thereby underscoring their potential as therapeutic targets. Neutrophils, particularly tumor-associated neutrophils (TANs), have emerged as critical contributors to cancer progression [2–4]. In addition, an increased neutrophil-to-lymphocyte ratio in peripheral blood is a well-defined predictive marker for a worse prognosis in several cancers, including CRC [5, 6].

Chemokines orchestrate infiltration and localization of various immune cells within TME via their receptors. We previously demonstrated that aberrations in TGF-β signaling caused by SMAD4 deficiency promoted the expression of mouse CCL9, or human CCL15 (a human ortholog of mouse CCL9) from CRC cells to recruit CCR1^+^ myeloid cells (TANs and MDSCs) within primary and metastatic CRCs [7–12]. Furthermore, inhibition of CCR1-mediated myeloid cell accumulation could be an effective therapeutic strategy in CRC mouse models [13]. Regarding a clinically applicable inhibitor of CCR1, we have recently established a neutralizing anti-CCR1 monoclonal antibody (mAb), KM5908, that could efficiently suppress the CCR1-mediated pathway in vitro and in vivo [13]. Meanwhile, there is emerging evidence for a tumor-promoting role of CXCR2 in the recruitment of neutrophils and MDSCs toward TME in several types of cancers [14–19]. We also demonstrated that recruitment of CXCR2^+^ myeloid cells to SMAD4-deficient CRCs could promote tumor invasion and metastasis, suggesting blockade of the CXCL1/8–CXCR2 axis could be a novel therapeutic approach in CRC [20]. Further, we found that myeloid cells positive for both CCR1 and CXCR2 were accumulated around the tip of the tumor in human clinical CRC specimens [7]. These results suggest that the two chemokine pathways, that is CCL9 (mouse)- or CCL15 (human)-CCR1 axis and CXCL1/8-CXCR2 axis, are critical for the myeloid cell accumulation toward CRC. However, the efficacy of the concurrent inhibition of CCR1 and CXCR2 for CRC treatment remains poorly documented. Therefore, in the present study, we focused on the possibility of dual blockade therapy of CCR1 and CXCR2 expressed on the myeloid cells.

## Materials and methods

### Animal (Mice)

CCR1-deficient mice (*Ccr1*^−/−^) on C57BL/6 background were previously described [9]. B6.129S2(C)-*Cxcr2*^tm1Mwm^/J mice, heterozygous for Cxcr2, were obtained from Jackson Laboratories (Bar Harbor, ME, USA). C57BL/6 mice double-knockout for CCR1 and CXCR2 (*Ccr1*^−/−^*Cxcr2*^−/−^) were respectively generated by crossing each heterozygous mice. All mice were housed in a SPF (Specific Pathogen Free) animal facility.

### Cell lines

Mouse colon cancer cell lines (MC38, MC38 with luciferase (MC38-luc), CT26, Colon-26, and CMT93) and rat normal intestinal epithelium cell line (IEC-6) were cultured at 37 °C in low glucose DMEM with 10% FBS and 1% penicillin/streptomycin mixture under 5% CO_2_.

### Subcutaneous transplanted tumor model

For a transplanted tumor model, MC38 cells (1.0 ×10^6^ cells) suspended with 50 μL PBS and 50 μL Matrigel (Corning, Somerville, MA, USA) were subcutaneously injected into the dorsal flanks of mice. The size of the transplanted subcutaneous tumors was measured as volume using the formula (L1 × L1 × L2)/2, where L1 is the shortest diameter and L2 is the longest diameter. On days 35–42 post-inoculation, mice were sacrificed, and the transplanted tumors were harvested for histological analyses.

### Experimental liver metastasis model

For an experimental liver metastasis model, MC38 cells (1.0 ×10^6^ cells) suspended in 100 μl PBS were injected into the hilum of the spleen of mice. The spleen was removed 1 min after tumor cell injection to prevent splenic tumor formation. For in vivo bioluminescence imaging, 1 mg of VivoGlo™ Luciferin (Promega) was injected intraperitoneally into tumor-bearing mice 10 min before imaging. Bioluminescence from the MC38-luc cells was monitored on days 1, 4, 7, 14, 21 post-injection, using a Xenogen IVIS system (Xenogen Corporation). On day 21 post-injection, the mice were euthanized, and the liver was harvested for histological analyses.

### Public database

Data from TCGA program related to 13 types cancers including colon adenocarcinoma (COAD) and rectal adenocarcinoma (READ) were obtained and analyzed by cBioPortal (https://www.cbioportal.org/) and GEPIA (Gene Expression Profiling Interactive Analysis) (http://gepia.cancer-pku.cn/index.html). For analyzing single-cell RNA sequencing data, we utilized the Human Colon Cancer Atlas (c295) available at the Single Cell Portal (https://singlecell.broadinstitute.org/single_cell).

### Patients’ population

Serum levels of CCL15, CXCL1, and CXCL8 were measured using preoperative serum samples collected from 94 patients with cStage I-III CRC between 2011 and 2018.

### Bone marrow (BM) transplantation

BM cells harvested from each donor mice were injected into the tail vein of recipient wild-type C57BL/6 mice that had been lethally irradiated with 9.5-Gy gamma-rays half a day before. After 12 weeks of BM transplantation, the recipient mice were inoculated with tumor cells. Recipient mice were treated prophylactically with antibiotic water (83 mg/L ciprofloxacin and 67 mg/L polymyxin B) for 7 days prior to the transplantation and during the entire duration of the experiments, as *Cxcr2*^−/−^ mice are reported to be susceptible to infections [21].

### Polymerase chain reaction (PCR)

Tail clippings were performed on 3–4-week-old mice in order to isolate genomic DNA and provide confirmation of the *Ccr1* and *Cxcr2* status. Tail tissues were digested at 95 °C for 10 min in NaOH (50 mM) lysis buffer and 50 mM Tris pH 8.0. Genomic DNA that was obtained after three phenol–chloroform extractions was resuspended in sterile water. Using a 35-cycle PCR, the genomic DNA was amplified with specific primers in an automatic temperature cycler. Primer sequences used for genotyping are shown in Supplementary Table 1. PCR amplification products were electrophoresed through a 1% agarose gel containing ethidium bromide.

### Quantitative RT-PCR

Total RNA was extracted using the High Pure RNA Isolation Kit (Roche Diagnostics). Complementary DNAs were generated using ReverTra ace qPCR RT kit (Toyobo Co. Ltd.,). Primer sequences used for RT-PCR are shown in Supplementary Table 2.

### Flow cytometric analysis

Flow cytometric analysis and cell sorting were performed to examine cells isolated from bone marrow, subcutaneous tumors, and liver metastases using BD FACS Aria II (BD Biosciences), as previously described [9]. Sample preparation procedures included subcutaneous tumors on days 14 and 21 post-inoculation and liver metastases on days 7 and 14 post-injection. Tumors were harvested, rinsed in cold PBS, minced into small pieces, and dissociated with Tumor Dissociation Kit (Miltenyi Biotec) and gentle MACS Dissociator (Miltenyi Biotec) according to the manufacturer’s protocols. Red blood cells were removed using Lysing Buffer (BD Biosciences) and samples were resuspended in PBS supplemented with 2% FBS. Cells were stained with anti-CD45 (clone 30-F11), anti-CD11b Ab (clone M1/70), anti-Ly6G Ab (clone 1A8), anti-Ly6C Ab (clone HK1.4), and anti-CXCR2 Ab (clone SA044G4) (Supplementary Table 3). Anti-CCR1 mAb, KM5908, was provided from Kyowa Kirin Co., Ltd [13]. Propidium iodide (PI) was used to eliminate dead cells. Data were analyzed with the FlowJo software (BD Biosciences).

### Histological analysis

The methodology involved the fixing of mouse samples in 4% paraformaldehyde and embedding in paraffin, with tissue sections being stained with hematoxylin & eosin (HE) and the corresponding primary antibodies for immunohistochemistry (IHC) (Supplementary Table 4). Antigen retrieval was performed for all antibodies used in this study. The tissue sections were treated with a citrate buffer (pH 9, Dako) and subjected to heat-induced epitope retrieval. Densities of Ly6G^+^, CD8^+^, FOXP3^+^, CCR1^+^ and CXCR2^+^ cells at the peritumoral region, and CD31^+^ endothelial cells within the tumors (as a measure of tumoral microvessel density) were quantified. Tissue slides were consecutively analyzed by the investigator blinded to the group allocation. Five random files from each sample were analyzed at 200× original magnification. For immunofluorescence analysis (IF), tissue sections following antigen retrieval were incubated with primary antibodies overnight at 4 °C, followed by the 2nd antibodies (Supplementary Table 5). Representative images of IHC and IF were captured using a microscope (BZ-X800; Keyence).

### Neutrophil isolation and transwell migration assay

After BM cells were harvested, neutrophils were isolated by positive selection using a Ly6G isolation kit (Miltenyi Biotec, UK). The Ly6G^+^ cells were resuspended into a buffer (0.5% BSA in RPMI 1640) of 10^7^ cells/ml. Neutrophil migration was examined in sterile polystyrene 24-well plates fitted with transwell-permeable supports that contained 3 μm pore-size polycarbonate membranes (Corning, Corning, NY, USA). Neutrophils (100 μl of the suspension) were added to the upper chamber and preincubated in the buffer at 37 °C for 60 min. In the lower chamber, MC38 cancer medium was added as a chemoattractant. A negative control was established by adding buffer to the lower chamber only. Subsequently, the chambers were co-incubated for 24 h at 37 °C in 5% CO_2_. Migrated neutrophils were recovered from the lower chamber and quantified by FACS Acuri.

### Statistical analysis

All results were confirmed using at least three independent experiments. Values were expressed as means ±standard error of the mean (SEM). The statistical significance of differences was determined by Student’s *t* test, Mann–Whitney *U* test or chi-square test. The log-rank test was used for analysis of overall survival (OS) and relapse-free survival (RFS). All analyses were 2-sided, and a *P* value of <0.05 was considered statistically significant. Statistical analyses were performed using JMP Pro software version 14.0 (SAS Institute, Cary, NC, USA).

## Results

### High expression of ligands for CXCR2 and CCR1 could be a biomarker of CRC patients with poor prognosis

**Fig. 1:**
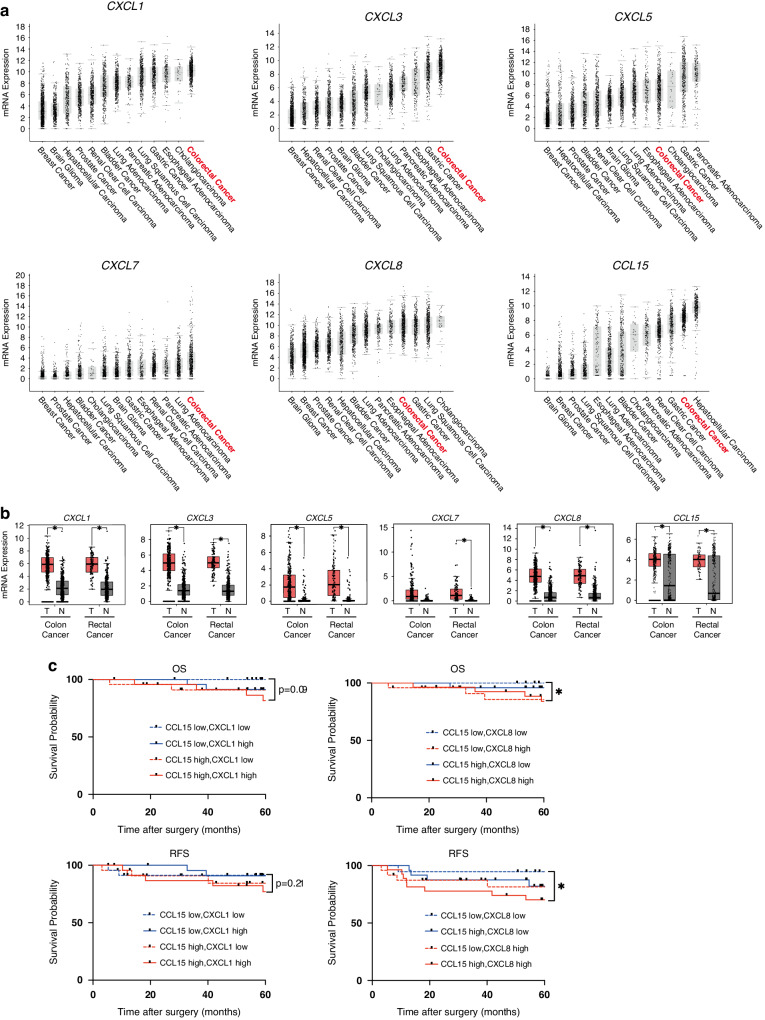
Expression of ligands for CXCR2 and CCR1 in CRC. **a** Plot illustrating expression levels of CXCR2 ligands (CXCL1, CXCL3, CXCL5, CXCL7 and CXCL8) and CCR1 ligand (CCL15) in various types of cancer from TCGA database. **b** Expression levels of CXCR2 ligands (CXCL1, CXCL3, CXCL5, CXCL7 and CXCL8) and CCR1 ligand (CCL15) in tumor tissues (red box) and adjacent normal tissues (gray box). *P < 0.01 by one-way ANOVA. T and N indicate tumor tissues and normal tissues. **c** Kaplan–Meier survival curves showing overall survival (OS) and relapse-free survival (RFS) in CRC patients, grouped by their serum levels of CXCL1, CXCL8, and CCL15. *P < 0.05 by log-rank test.

We first explored the expression of ligands for CCR1 and CXCR2 using The Cancer Genome Atlas (TCGA) database (COAD and READ). TCGA database indicated that among several ligands, CXCL1, CXCL3, CXCL5, CXCL7 and CXCL8 (ligands for CXCR2) and CCL15 (ligand for CCR1) were particularly upregulated in CRC tissues compared with other cancers (Fig. 1a and Supplementary Fig. 1a). Moreover, most of these ligands were significantly upregulated in cancer tissues than in adjacent normal tissues (Fig. 1b). Among the CXCR2 ligands, especially CXCL1 and CXCL8 have often been reported to be associated with cancer progression [22, 23].

Further, we investigated whether the serum levels of these ligands for CXCR2 and CCR1 (CXCL1, CXCL8 and CCL15) could be prognostic markers of CRC progression. We quantified the concentrations of CXCL1, CXCL8 and CCL15 in serum samples collected from patients with cStage IｰIII CRC (*n* = 94), and then classified them into 4 groups based on CXCL1 or 8 level and CCL15 level (Fig. 1c). Statistical analysis revealed that both OS and RFS were significantly lower in the patients with high CXCL8 and high CCL15 than in those with low CXCL8 and low CCL15 (log-rank test; *P* = 0.039 and 0.049, respectively). Patients with high CXCL1 and high CCL15 tended to exhibit a shorter OS and RFS than those with low CXCL1 and low CCL15 (*P* = 0.09 and 0.21, respectively), although the difference was not statistically significant. From these results, high expression of ligands for CCR1 and CXCR2 could be a biomarker for CRC patients with poor prognosis.

### Myeloid cell accumulation toward CRC tumors through CCR1- and CXCR2-mediated pathways

**Fig. 2:**
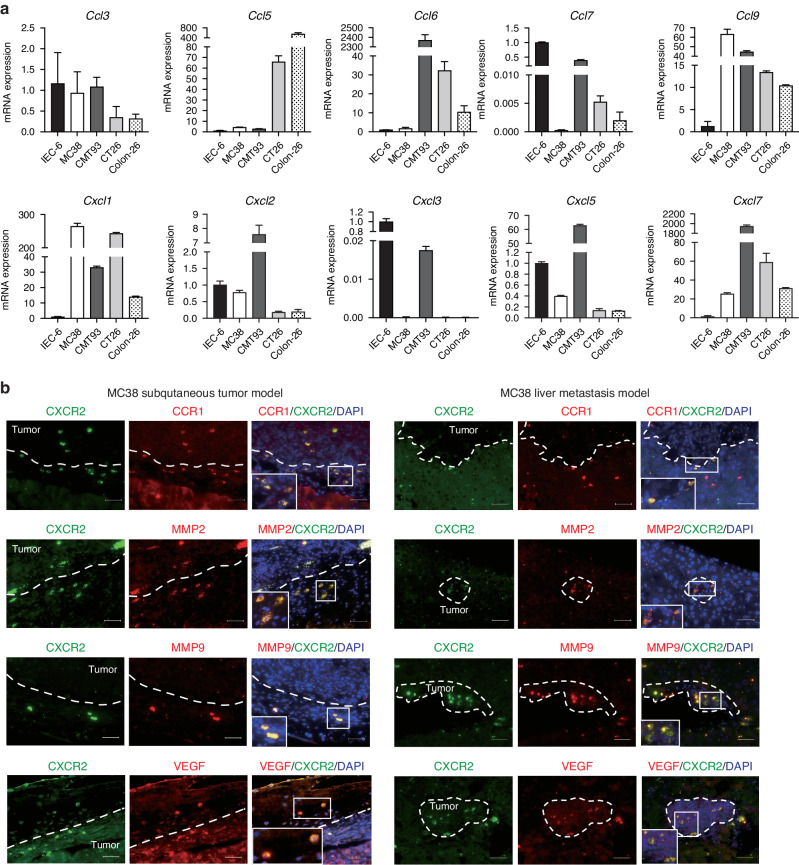
Accumulation of CCR1^+^CXCR2^+^ myeloid cells around CRC tumors. **a** mRNA expression levels of mouse CXCR2 ligands (CXCL1, CXCL2, CXCL3, CXCL5 and CXCL7) and mouse CCR1 ligands (CCL3, CCL5, CCL6, CCL7 and CCL9) in mouse colon cancer cell lines (MC38, CMT93, CT26 and Colon-26) and rat normal intestinal cell line (IEC-6). Although the species were different, primers with good amplification were used. **b** Simultaneous immunofluorescence staining for CXCR2 (green) and CCR1, MMP2, MMP9 or VEGF (red). Scale bar, 20 mm.

Although we previously demonstrated that disruption of CCR1-mediated myeloid cell accumulation suppressed tumor progression in syngeneic mouse models [13], it remains unclear whether disruption of CXCR2-mediated myeloid cell accumulation can suppress tumor progression. Therefore, we investigated the role of CXCR2 in myeloid cells using two syngeneic CRC mouse models of MC38 cells: a transplanted tumor model and a liver metastasis model. We initially measured the mRNA expression levels of ligands for CXCR2 and CCR1 in mouse colon cancer cell lines (MC38, CMT93, CT26 and Colon-26) and a normal rat intestinal cell line (IEC-6) (Fig. 2a). Among the several CCR1 ligands, *Ccl9* mRNA was abundantly produced in all mouse colon cancer cell lines, although few levels of *Ccl9* mRNA were detected in IEC-6 cells. Among the several CXCR2 ligands, *Cxcl1* mRNA was abundantly produced in all mouse colon cancer cell lines, although few levels of *Cxcl1* mRNA were detected in IEC-6 cells. In these MC38 tumor models, CXCR2^+^ myeloid cells were accumulated around the tumors in wild-type C57BL/6 mice (Fig. 2b). We also characterized CXCR2^+^ myeloid cells by double immunofluorescence staining, and found that majority of these CXCR2^+^ cells were also positive for CCR1, and expressed matrix metalloproteinase (MMP) 2, MMP 9 and vascular endothelial growth factor (VEGF) in both the transplanted tumors and liver metastases (Fig. 2b). Interestingly, when human CCR1 and CXCR2 were examined, data from a public database of single-cell data from human colorectal cancers (https://singlecell.broadinstitute.org/single_cell) revealed the presence of myeloid cells positive for both CCR1 and CXCR2 at the primary site of colorectal cancer (Supplementary Fig. 1b). This observation underscores the relevance of our findings in mouse models to the human condition, indicating a conserved mechanism of myeloid cell accumulation that may contribute to cancer progression and serve as a target for therapeutic intervention.

### Knockout of CXCR2 results in suppression of CRC progression

**Fig. 3:**
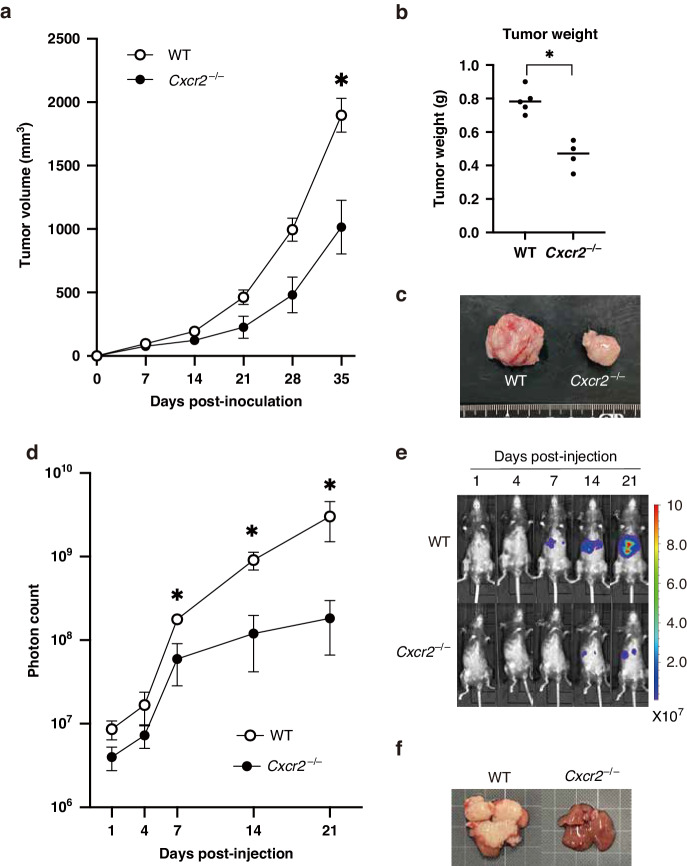
Lack of CXCR2 in host mice suppresses CRC tumor growth and metastasis. **a** Tumor growth curves of transplanted MC38 tumors in wild-type and *Cxcr2*^−/−^ mice. Bars, mean ± SEM (Student’s *t* test; *, *P* < 0.05). *n* = 4–5 mice for each group. **b** Tumor weight of transplanted MC38 tumors in wild-type and *Cxcr2*^−/−^ mice on day 35 post-injection. *P < 0.05 by Student’s *t* test. **c** Representative macroscopic views of transplanted MC38 tumors in wild-type and *Cxcr2*^−/−^ mice on day 35 post-injection. **d** Quantification of liver metastatic lesions (photon counts). Bars, mean ± SEM (Mann–Whitney *U* test; *P < 0.05). *n* = 4–5 mice for each group. **e** Representative in vivo bioluminescence images of MC38-luc liver metastases in wild-type and *Cxcr2*^−/−^ mice. **f** Representative macroscopic views of the livers dissected from wild-type and *Cxcr2*^−/−^ mice on day 21 post-injection. Note that only wild-type mice exhibited large metastatic foci.

Next, we investigated the effect of CXCR2 deletion of the host mice in MC38 tumor models. As a transplanted tumor model, we injected MC38 cells subcutaneously into wild-type or *Cxcr2*^−/−^ mice. The growth of MC38 transplanted tumors was significantly reduced in *Cxcr2*^−/−^ mice compared with wild-type mice (Fig. 3a–c). On day 35 post-injection, the tumor size and weight in wild-type mice were 1897 ± 133 mm^3^ and 0.786 ± 0.01 g, whereas those in *Cxcr2*^−/−^ mice were 1015 ± 211 mm^3^ and 0.46 ± 0.04 g (*P* < 0.05 and <0.05, respectively).

As a model of liver metastasis, we injected MC38-luc cells into the spleen of wild-type or *Cxcr2*^−/−^ mice, and then monitored liver metastasis using bioluminescence to quantify tumor cells within the liver. Liver luciferase intensities in *Cxcr2*^−/−^ mice were significantly lower compared with those in wild-type mice (Fig. 3d, e). On day 21 post-injection, there was a significant reduction of the liver luminescence in *Cxcr2*^−/−^ mice compared with that in wild-type mice (mean, 1.57 × 10^8^ vs. 3.28 ×10^9^; *P* < 0.05), which occurred only after day 7 post-injection. We dissected the liver on day 21 post-injection, and then confirmed that macroscopic large metastatic foci existed only in wild-type mice (Fig. 3f), consistent with the bioluminescence analysis.

### Lack of CXCR2 in hematopoietic myeloid cells leads to suppression of CRC development

**Fig. 4:**
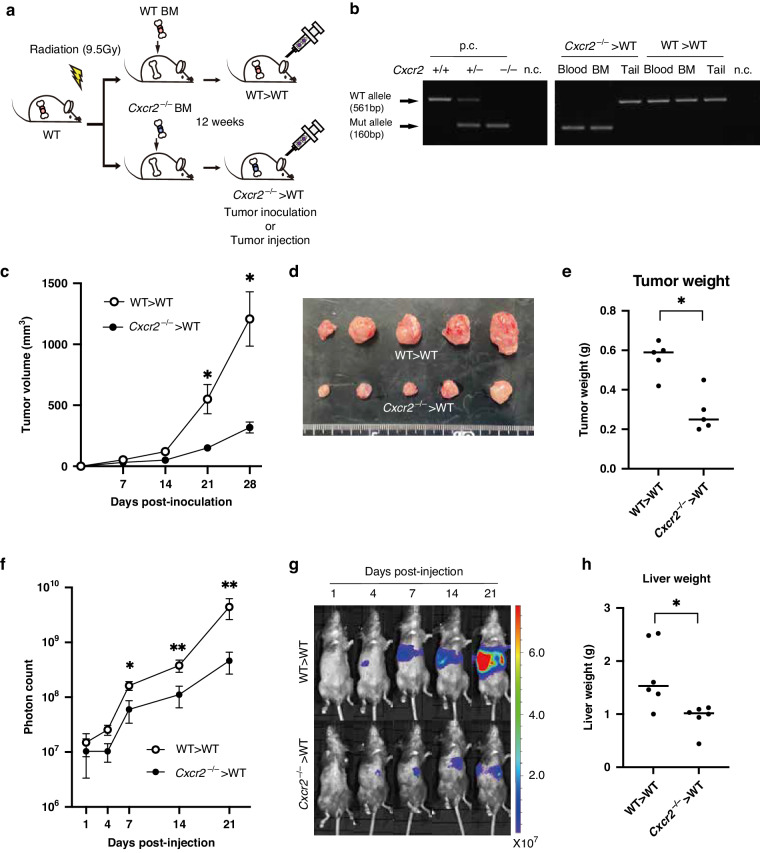
Lack of CXCR2 in hematopoietic myeloid cells suppresses CRC tumor growth and metastasis. **a** Scheme of BM transfer experiments. Wild-type recipient hosts were sub-lethally irradiated and then reconstituted with wild-type BM (WT > WT mice) or *Cxcr2*^−/−^ BM (*Cxcr2*^−/−^ > WT mice). **b** PCR analysis of wild-type (561 kb) and mutated allele (160 kb) of *Cxcr2*. p.c., positive control. n.c. negative control. **c** Tumor growth curves of transplanted MC38 tumors in WT > WT mice and *Cxcr2*^−/−^ > WT mice. Bars, mean ± SEM (Student’s *t* test; *P < 0.05). *n* = 5 mice for each group. **d** Representative macroscopic views of transplanted MC38 tumors in WT > WT mice and *Cxcr2*^−/−^> WT mice. **e** Tumor weight of transplanted MC38 tumors in WT > WT mice and *Cxcr2*^−/−^>WT mice on day 28 post-inoculation. *P < 0.05 by Student’s *t* test. **f** Quantification of liver metastatic lesions (photon counts). Bars, mean ± SEM (Mann–Whitney *U* test; *P < 0.05 and **P < 0.01). *n* = 6 mice for each group. **g** Representative in vivo bioluminescence images of MC38-luc liver metastases in WT > WT mice and *Cxcr2*^−/−^ > WT mice. **h** Liver weight of WT > WT mice and *Cxcr2*^−/−^ > WT mice on day 21 post-injection. *P < 0.05 by Student’s *t* test.

Given that CXCR2 is detectable in endothelial cells [14], we performed BM transfer experiments to evaluate the contribution of CXCR2^+^ myeloid cells. Namely, sub-lethally irradiated wild-type mice were reconstituted with BM derived from either wild-type or *Cxcr2*^−/−^ mice, and then inoculated with MC38 cells (Fig. 4a). We confirmed that chimeric mice reconstituted with *Cxcr2*^−/−^ BM (*Cxcr2*^−/−^ > WT mice) exhibited a depletion of circulating CXCR2^+^ myeloid cells (Fig. 4b). We evaluated the growth kinetics of MC38 transplanted tumors, and found that *Cxcr2*^−/−^ > WT mice exhibited significantly smaller tumors compared with recipient mice of wild-type BM (WT > WT mice) (Fig. 4c–e). On day 28 post-injection, the tumor size and weight in *Cxcr2*^−/−^ > WT mice were 318 ± 45 mm^3^ and 0.284 ± 0.04 g, whereas those in WT > WT mice were 1208 ± 223 mm^3^ and 0.562 ± 0.04 g (*P* < 0.05 and <0.05, respectively).

Next, we administered MC38-luc cells into the spleen of either WT > WT mice or *Cxcr2*^−/− ^> WT mice, and then monitored the metastasized cells within the liver through in vivo bioluminescence (Fig. 4f, g). On days 21 post-injection, *Cxcr2*^−/−^ > WT mice exhibited significantly lower photon counts in the liver compared with WT > WT mice (mean, 4.63 × 10^8^ vs. 4.42 × 10^9^; *P* < 0.05), which occurred only after day 7 post-injection. We dissected the liver on day 21 post-injection, and found that macroscopic foci were observed in 50% (3 of 6) of *Cxcr2*^−/−^ > WT mice, whereas observed in 100% (6 of 6) of WT > WT mice (*P* < 0.01; Supplementary Fig. 2a). We also confirmed that the liver weight of *Cxcr2*^−/−^ > WT mice was significantly lower compared with that in WT > WT mice (Fig. 4h).

### Dual blockade of CCR1 and CXCR2 in hematopoietic myeloid cells dramatically halts myeloid cell accumulation and tumor progression

**Fig. 5:**
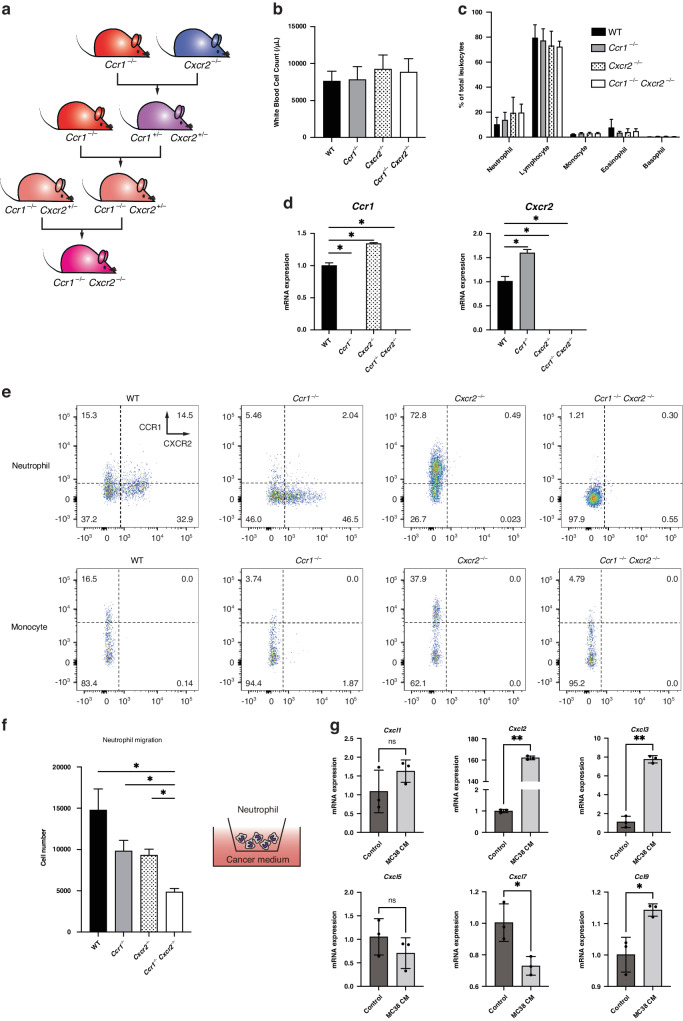
Construction of double-knockout mice for both CCR1 and CXCR2 (*Ccr1*^−/−^*Cxcr2*^−/−^ mice). **a** Scheme of the breeding strategy to construct *Ccr1*^−/−^*Cxcr2*^−/−^ double-knockout mice. **b** Total white blood cell count in peripheral blood. *n* = 5 mice for each group. **c** Distribution of total leukocytes in peripheral blood. **d** Expression levels of CCR1 and CXCR2 in myeloid cells. *P < 0.01 by Student’s *t* test. *n* = 3 mice for each group. **e** Flow cytometric analysis of CCR1 and CXCR2 expression in BM cells. **f** In vitro migration assay of neutrophils toward the supernatant of MC38 cancer cells. *P < 0.05 by Student’s *t* test. **g** Expression of CXCR2 ligands (CXCL1, CXCL2, CXCL3, CXCL5 and CXCL7) and CCR1 ligand (CCL9) in neutrophils cocultured with the supernatant of MC38 cancer cells. *P < 0.05 and **P < 0.01 by Student’s *t* test.

To investigate whether the dual blockade of CCR1 and CXCR2 has an additional effect in recruiting myeloid cells toward CRC tumors compared with the single blockade of each receptor, we generated double-knockout mice for CCR1 and CXCR2 (*Ccr1*^−/−^*Cxcr2*^−/−^) by crossing *Ccr1*^−/−^ mice and *Cxcr2*^−/−^ mice (Fig. 5a and Supplementary Fig. 2b). There were no significant differences in the cell number and distribution of circulating blood cells among wild-type, *Ccr1*^−/−^, *Cxcr2*^−/−^ and *Ccr1*^−/−^*Cxcr2*^−/−^ double-knockout mice (Fig. 5b, c). Regarding the chemokine receptors in myeloid cells, we found that mRNA level of CCR1 was significantly upregulated in *Cxcr2*^−/−^ mice than in wild-type mice, and that mRNA level of CXCR2 was significantly upregulated in *Ccr1*^−/−^ mice than in wild-type mice (Fig. 5d), indicating a compensatory mechanism. We also evaluated the expression levels of CCR1 and CXCR2 in wild-type BM cells using flow cytometry, and found that neutrophils (CD45^+^CD11b^+^Ly6C^mid^Ly6G^high^) expressed high levels of CXCR2 and moderate levels of CCR1, whereas monocytes (CD45^+^CD11b^+^Ly6C^high^Ly6G^−^) expressed only CCR1 (Fig. 5e, left). Neutrophils were a heterogenous population in terms of expression of CCR1 and CXCR2, while monocytes were a heterogenous population in terms of CCR1 expression. Flow cytometric analysis confirmed that a single knockout of CCR1 or CXCR2 resulted in the loss of each receptor, and a double-knockout of CCR1 and CXCR2 resulted in the lack of both receptors (Fig. 5e, middle and right). Furthermore, CCR1 expression was much higher in *Cxcr2*^−/−^ mice than in wild-type mice, whereas CXCR2 expression was a little higher in *Ccr1*^−/−^ mice than in wild-type mice, which was almost consistent with the data observed in Fig. 5d (Fig. 5e, middle). To elucidate the migratory response of neutrophils, we further conducted an in vitro migration assay using neutrophils isolated from the BM cells of WT, *Ccr1*^−/−^, *Cxcr2*^−/−^ and *Ccr1*^−/−^*Cxcr2*^−/−^ mice. To simulate a biological situation, neutrophils were added to the upper chamber, while the supernatant of MC38 cancer medium was added to the lower chamber (Fig. 5f and Supplementary Fig. 3). As expected, the migratory response of neutrophils from *Ccr1*^−/−^ or *Cxcr2*^−/−^ mice was significantly suppressed compared with that from WT mice. Importantly, neutrophils from *Ccr1*^−/−^*Cxcr2*^−/−^ mice exhibited the lowest migration. We also measured the mRNA levels of ligands for CCR1 and CXCR2 in neutrophils from WT mice by RT-qPCR. Of note, CXCL2, CXCL3, and CCL9 were markedly increased when neutrophils were co-cultured with the supernatant of MC38 cells, which might lead to a positive feedback loop that perpetuates granulocyte stimulation (Fig. 5g).

**Fig. 6:**
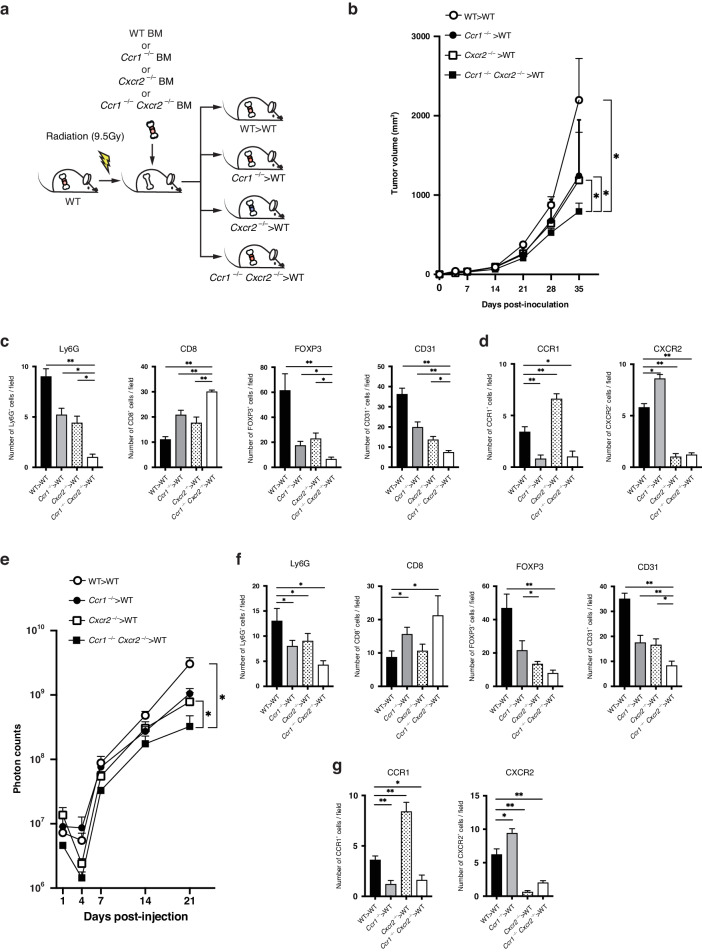
Lack of both CCR1 and CXCR2 in hematopoietic myeloid cells dramatically suppresses CRC tumor growth and metastasis. **a** Scheme of BM transfer experiments. Wild-type recipient hosts were sub-lethally irradiated and then reconstituted with wild-type BM (WT > WT mice), *Ccr1*^−/−^ BM (*Ccr1*^−/−^ > WT mice), *Cxcr2*^−/−^ BM (*Cxcr2*^−/−^ > WT mice) or *Ccr1*^−/−^*Cxcr2*^−/−^ BM (*Ccr1*^−/−^*Cxcr2*^−/−^ > WT mice). **b** Tumor growth curves of transplanted MC38 tumors in WT > WT mice, *Ccr1*^−/−^ > WT mice, *Cxcr2*^−/−^ > WT mice and *Ccr1*^−/−^*Cxcr2*^−/−^ > WT mice. Bars, mean ± SEM (Student’s *t* test; *P < 0.05). *n* = 5 mice for each group. **c** Immunohistological staining for Ly6G^+^ neutrophils, CD8^+^ T cells, FOXP3^+^ Treg cells and CD31^+^ endothelial cells around transplanted MC38 tumors. *P < 0.05 and **P < 0.01 by Student’s *t* test. **d** Immunohistological staining for CCR1^+^ and CXCR2^+^ cells. *P < 0.05 and **P < 0.01 by Student’s *t* test. **e** Quantification of liver metastatic lesions (photon counts). Bars, mean ± SEM (Mann–Whitney *U* test; *P < 0.05). *n* = 3–6 mice for each group. **f** Immunohistological staining for Ly6G^+^ neutrophils, CD8^+^ T cells, FOXP3^+^ Treg cells and CD31^+^ endothelial cells around MC38 liver metastases. *P < 0.05 and **P < 0.01 by Student’s *t* test. **g** Immunohistological staining for CCR1^+^ and CXCR2^+^ cells. *P < 0.05 and **P < 0.01 by Student’s *t* test.

To elucidate the contribution of CCR1^+^ CXCR2^+^ myeloid cells in CRC progression, we further performed BM transfer experiments using 4 groups: wild-type BM, *Ccr1*^−/−^ BM, *Cxcr2*^−/−^ BM and *Ccr1*^−/−^*Cxcr2*^−/−^ BM (Fig. 6a). In the transplanted tumor model, mice reconstituted with *Ccr1*^−/−^*Cxcr2*^−/−^ BM (*Ccr1*^−/−^*Cxcr2*^−/−^ > WT mice) exhibited dramatically smaller tumors compared with recipients of wild-type BM (WT > WT mice), *Ccr1*^−/−^ BM (*Ccr1*^−/−^ > WT mice) and *Cxcr2*^−/−^ BM (*Cxcr2*^−/−^ > WT mice). On day 35 post-inoculation, the tumor size in *Ccr1*^−/−^*Cxcr2*^−/−^ > WT mice was 794 ± 104 mm^3^, whereas those in WT > WT, *Ccr1*^−/−^>WT and *Cxcr2*^−/−^ > WT mice were 2198 ± 525 mm^3^, 1243 ± 352 mm^3^ and 1188 ± 246 mm^3^ (*P* < 0.05, <0.05 and <0.05, respectively: Fig. 6b and Supplementary Fig. 4b). No differences in body weight of mice were observed between the groups over time (Supplementary Fig. 4a). To investigate the impact on immune infiltrating cells, we quantified the density of CD8^+^ cytotoxic T cells, FOXP3^+^ regulatory T (Treg) cells, CD31^+^ endothelial cells and Ly6G^+^ neutrophils around the tumors. Immunohistological analysis indicated that, in *Ccr1*^−/−^*Cxcr2*^−/−^ > WT mice, the number of CD8^+^ T cells was significantly higher, while the numbers of Ly6G^+^ neutrophils, FOXP3^+^ Treg cells and CD31^+^ endothelial cells were markedly lower compared with the recipient mice of the other three groups (Fig. 6c and Supplementary Fig. 4d). We also quantified the density of CCR1^+^ cells and CXCR2^+^ cells around the tumors. The number of CCR1^+^ cells in *Cxcr2*^−/−^ > WT mice was significantly higher than that in WT > WT mice, whereas the number of CXCR2^+^ cells in *Ccr1*^−/−^ > WT mice was significantly higher than that in WT > WT mice (Fig. 6d and Supplementary Fig. 4d). We further characterized the infiltrating myeloid cells using flow cytometry analysis (Supplementary Fig. 5a). Transplanted tumors from *Ccr1*^−/−^*Cxcr2*^−/−^ mice on day 21 post-inoculation showed the greatest reduction in both granulocytic MDSCs (CD11b^+^Ly6C^mid^Ly6G^high^ cells) and monocytic MDSCs (CD11b^+^Ly6C^high^Ly6G^−^ cells) compared to the other three groups, which was not observed on day 14 post-inoculation (Supplementary Fig. 5b, d).

In the liver metastasis model, depletion of both CCR1 and CXCR2 in myeloid cells also resulted in significant suppression of liver metastasis (Fig. 6e and Supplementary Fig. 4c). On day 21 post-injection, liver luminescence in *Ccr1*^−/−^*Cxcr2*^−/−^ > WT mice were significantly lower compared with those in WT > WT, *Ccr1*^−/−^ > WT and *Cxcr2*^−/−^ > WT mice (mean, 3.25 ×10^8^ vs. 3.05 ×10^9^, *P* < 0.05; vs. 1.05 ×10^9^, *P* = 0.098 and vs. 7.82 ×10^8^, *P* < 0.05, respectively). We also quantified the immune infiltrating cells within liver metastases, and found that depletion of CCR1^+^ CXCR2^+^ myeloid cells led to a higher frequency of CD8^+^ T cells, whereas the numbers of Ly6G^+^ neutrophils, FOXP3^+^ Treg cells and CD31^+^ endothelial cells were significantly decreased (Fig. 6f and Supplementary Fig. 4e). The number of CCR1^+^ cells in *Cxcr2*^−/−^ > WT mice was significantly higher than that in WT > WT mice, whereas the number of CXCR2^+^ cells in *Ccr1*^−/−^ > WT mice was significantly higher than that in WT > WT mice (Fig. 6g and Supplementary Fig. 4e). Metastatic tumors from *Ccr1*^−/−^*Cxcr2*^−/−^ mice on day 14 post-injection showed the greatest reduction in both granulocytic MDSCs (CD11b^+^Ly6C^mid^Ly6G^high^ cells) and monocytic MDSCs (CD11b^+^Ly6C^high^Ly6G^−^ cells) compared to the other three groups, which was not observed on day 7 post-injection (Supplementary Fig. 5c, e). These experimental results suggest that dual blockade of CCR1 and CXCR2 pathways in myeloid cells can cause effective antitumor activity in CRC progression.

### Synergistic effects of a novel anti-CCR1 mAb on genetic CXCR2 knockout

As a clinically applicable CCR1 inhibitor, we previously established a novel neutralizing anti-CCR1 mAb, KM5908, which was proven to suppress CCR1^+^ myeloid cell accumulation and tumor progression in preclinical mouse models [13]. Therefore, we further examined whether the dual blockade of CCR1 and CXCR2 by KM5908 and genetic CXCR2 knockout could exhibit a synergistic effect.

**Fig. 7:**
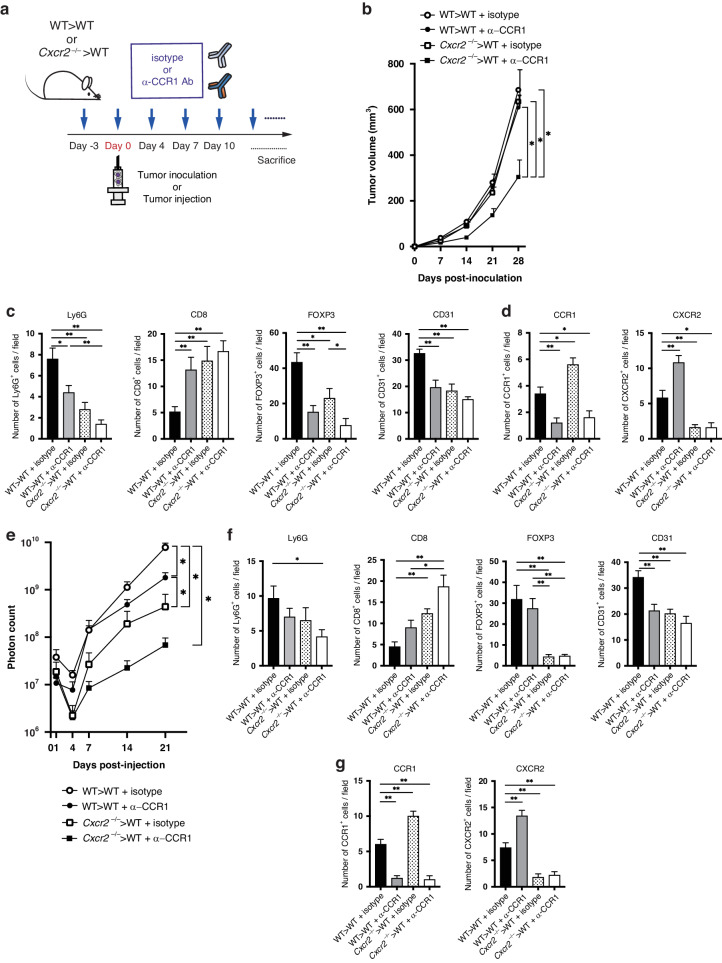
Effect of anti-CCR1 mAb, KM5908, on tumor growth and liver metastasis in the context of genetic CXCR2 knockout. **a** Experimental scheme of KM5908 or isotype control administration in WT > WT mice and *Cxcr2*^−/−^ > WT mice. **b** Tumor growth curves of transplanted MC38 tumors in the four treatment groups: isotype-treated WT > WT mice, KM5908-treated WT > WT mice, isotype-treated *Cxcr2*^−/−^ > WT mice and KM5908-treated *Cxcr2*^−/−^ > WT mice. Bars, mean ± SEM (Student’s *t* test; *P < 0.05). *n* = 4–5 mice for each group. **c** Immunohistological staining for Ly6G^+^ neutrophils, CD8^+^ T cells, FOXP3^+^ Treg cells and CD31^+^ endothelial cells around transplanted MC38 tumors. Scale bar, 100 mm. *P < 0.05 and **P < 0.01 by Student’s *t* test. **d** Immunohistological staining for CCR1^+^ and CXCR2^+^ cells. *P < 0.05 and **P < 0.01 by Student’s *t* test. **e** Quantification of liver metastatic lesions (photon counts) in the four treatment groups: isotype-treated WT > WT mice, KM5908-treated WT > WT mice, isotype-treated *Cxcr2*^−/−^ > WT mice and KM5908-treated *Cxcr2*^−/−^ > WT mice. Bars, mean ± SEM (Mann–Whitney *U* test; *P < 0.05). *n* = 3–6 mice for each group. **f** Immunohistological staining for Ly6G^+^ neutrophils, CD8^+^ T cells, FOXP3^+^ Treg cells and CD31^+^ endothelial cells around transplanted MC38 tumors. *P < 0.05 and **P < 0.01 by Student’s *t* test. **g** Immunohistological staining for CCR1^+^ and CXCR2^+^ cells. **P < 0.01 by Student’s *t* test.

We administered 10 μg/g KM5908 or isotype control to WT > WT mice or *Cxcr2*^−/−^ > WT mice, and assessed its efficacy in vivo (Fig. 7a). In the MC38 transplanted tumor model, tumor growth was significantly suppressed in *Cxcr2*^−/−^ > WT mice treated with KM5908 compared with that in the other 3 groups (Fig. 7b and Supplementary Fig. 6b). On day 28 post-injection, the tumor size in *Cxcr2*^−/−^ > WT mice treated with KM5908 was 304 ± 75 mm^3^, whereas those in WT > WT mice treated with isotype, WT > WT mice treated with KM5908 and *Cxcr2*^−/−^ > WT mice treated with isotype were 686 ± 88 mm^3^, 608 ± 53 mm^3^ and 634 ± 140 mm^3^, respectively. No differences in body weight of mice were observed between the groups over time (Supplementary Fig. 6a). Immunohistochemical analysis revealed that, in *Cxcr2*^−/−^ > WT mice treated with KM5908, the number of CD8^+^ T cells was significantly higher, while the numbers of Ly6G^+^ neutrophils, FOXP3^+^ Treg cells and CD31^+^ endothelial cells were significantly lower compared with those in the other three groups (Fig. 7c and Supplementary Fig. 6d). The number of CCR1^+^ cells in *Cxcr2*^−/−^ > WT mice treated with isotype was significantly higher than that in WT > WT mice treated with isotype, whereas the number of CXCR2^+^ cells in WT > WT mice treated with KM5908 was significantly higher than that in WT > WT mice treated with isotype (Fig. 7d and Supplementary Fig. 6d).

In the liver metastasis model, *Cxcr2*^−/−^>WT mice treated with KM5908 exhibited significantly reduced metastasized cells within the liver compared with the other three groups (Fig. 7e and Supplementary Fig. 6c). On day 21 post-injection, liver luminescence in KM5908-treated *Cxcr2*^−/−^ > WT mice was significantly lower than that in both isotype-treated and KM5908-treated WT > WT mice (mean, 6.83 ×10^7^ vs. 7.84 ×10^9^, *P* < 0.05 and vs. 1.80 ×10^9^, *P* < 0.05, respectively). There was also a reduction of liver luminescence in KM5908-treated *Cxcr2*^−/−^ > WT mice compared with that in isotype-treated *Cxcr2*^−/−^ > WT mice, although not a significant difference (mean, 6.83 × 10^7^ vs. 4.37 × 10^8^, *P* = 0.08). As anticipated, immunohistochemical analysis indicated that KM5908 treatment led to a significantly higher frequency of CD8^+^ T cells in *Cxcr2*^−/−^ > WT mice, whereas the numbers of Ly6G^+^ neutrophils, FOXP3^+^ Treg cells and CD31^+^ endothelial cells were significantly decreased (Fig. 7f and Supplementary Fig. 6e). The number of CCR1^+^ cells in *Cxcr2*^−/−^ > WT mice treated with isotype was significantly higher than that in WT > WT mice treated with isotype, whereas the number of CXCR2^+^ cells in WT > WT mice treated with KM5908 was significantly higher than that in WT > WT mice treated with isotype (Fig. 7g and Supplementary Fig. 6e). These results on preclinical models suggest that administration of the CCR1 inhibitor, KM5908, may have clinical applications.

## Discussion

Regarding the gene-chemokine relationship in CRC, we have shown that the loss of SMAD4 leads to the recruitment of myeloid cells via two critical pathways: CXCL1/8-CXCR2 axis and CCL9 (mouse)- or CCL15 (human)-CCR1 axis, promoting CRC progression [7–13, 20]. Similarly, KRAS mutations, another prominent genetic alteration in CRC, can lead to increased expression of CXCL3, thereby enhancing the migration of CXCR2^+^ MDSCs and fostering resistance to anti-PD-1 immunotherapy [15]. Recent studies have discovered that stem cell markers, such as doublecortin-like kinase (DCLK1) and RNA modification by methyltransferase-like 3 (METTL3), play a role in recruitment of CXCR2^+^ MDSCs to modulate tumor immunity through the CXCL1-CXCR2 axis in CRC mouse models [16, 17]. The CXCLs (i.e., CXCL1, 2, 3, 5, 7 and 8)-CXCR2 signaling axis is involved in the recruitment of MDSCs in various types of cancers, including CRC [18, 19, 22]. Neutrophils and MDSCs predominantly express CXCR2 and play crucial roles in their mobilization and subsequent tumor-associated activities [16, 24, 25]. Furthermore, CXCR2 ligands could induce neutrophil extracellular traps (NETs)-related activation and proliferation of dormant cancer cells [22, 26–29].

CCR1 is widely expressed on myeloid cells, including monocytes, macrophages and dendritic cells [30, 31]. CCR1 is also expressed on human neutrophils after stimulation of inflammatory mediators [32, 33]. In transgenic *Apc*^*+/∆716*^*/Smad4*^*+/-*^ compound knockout mice that develop CRC, CCL9 is secreted from cancer cells, which recruits CCR1^+^ myeloid cells to promote tumor invasion [10]. In a liver metastasis model, CCL9-expressing CRC cells recruit CCR1^+^ myeloid cells to expand liver metastases [11], and four different types of myeloid cells (i.e., CCR1^+^ neutrophils, monocytes, eosinophils and fibrocytes) are recruited to the liver metastases [9]. Several studies have also shown that the recruitment of CCR1^+^ myeloid cells via CCR1 ligands, such as CCL2, CCL9 and CCL15, promotes invasion, metastasis, and angiogenesis in CRC and other tumor types [8, 13, 34–37]. Various chemokine receptors, including CCR1, CXCR2, CCR2 and CXCR4, play crucial roles in immune cell trafficking and recruitment, particularly under inflammatory and cancerous conditions [38]. Interestingly, previous studies reported that that disrupting myeloid cell recruitment by jointly blocking CXCR2 and CCR2 could enhance anti-tumor immunity and therapeutic responses in pancreatic cancer models [39]. In the present study, CRC patients with high serum levels of CXCL1/8 and CCL15 had the poorest prognosis across cStage I − III (Fig. 1c). Furthermore, CCR1^+^ and CXCR2^+^ myeloid cells were concentrated around the tumor invasion front in our mouse model, which is consistent with a previous human CRC study [7] and data from a public database from human colorectal cancers (Supplementary Fig. 1b). Therefore, we investigated the effects of dual blockade of CCR1 and CXCR2 in a CRC mouse model. Double-knockout mice for CCR1 and CXCR2 have been studied for research on arthritis. In a mouse model of arthritis, CCR1 increased neutrophil crawling on the endothelium, while CXCR2 increased neutrophil retention and survival within the joints [40, 41]. However, studies using double-knockout mice for CCR1 and CXCR2 remain unexplored in cancer research. In the present study, we used double-knockout mice for CCR1 and CXCR2 in BM transfer experiments (Figs. 5 and 6), and discovered that simultaneous deletion of both CCR1 and CXCR2 entirely inhibited neutrophil mobilization, thereby significantly suppressing tumor growth and metastasis. The presence of chemokine receptor redundancy was corroborated by mRNA, flow cytometry and immunohistochemical staining results (Figs. 5d, e, 6d, g and 7d, g), where inhibition of one receptor is compensated by upregulation of another, supporting the rationale that dual blockade can be most effective.

For clinical applications, we utilized the anti-CCR1 mAb, KM5908, which has been proven to suppress the accumulation of CCR1^+^ myeloid cells in vivo [13]. Importantly, we could confirm a synergistic effect when KM5908 was used in conjunction with genetic CXCR2 knockout (Fig. 7). This co-treatment displayed a similar trend to the double-knockout mice for CCR1 and CXCR2, further supporting the potential of dual blockade therapy against CCR1 and CXCR2.

Recently, other new CCR1 antagonists have been reported in preclinical studies; for example, J-113863 showed efficacy in animal models of metastatic melanoma, multiple sclerosis and autoimmune encephalomyelitis, while BX471 was tested in models of asthma [37, 42–44]. It was recently reported that a conformational change of Tyr291 in CCR1 triggered its polar network rearrangement to regulate β-arrestin signaling [45], which can contribute to the development of new CCR1-targeted drugs. These advances in the field of chemokine receptor antagonists can further strengthen the rationale for their application in CRC treatment. CXCR2 inhibitors such as Navarixin, SB225002, SB265610 and AZD5069 have also been reported in preclinical studies. Moreover, these agents can improve immune checkpoint inhibition by reducing the accumulation of immunosuppressive MDSCs and promoting the infiltration of cytotoxic CD8^+^T cells in many cancer models, including rhabdomyosarcoma, NASH-HCC, CRC, PDAC, and lung cancer [15–17, 19, 46–49]. An ongoing clinical trial, NCT04599140, is examining the potential benefits of combined treatment with nivolumab and the CXCR1/2-receptor antagonist SX-682 in patients with microsatellite-stable CRC [50].

**Fig. 8:**
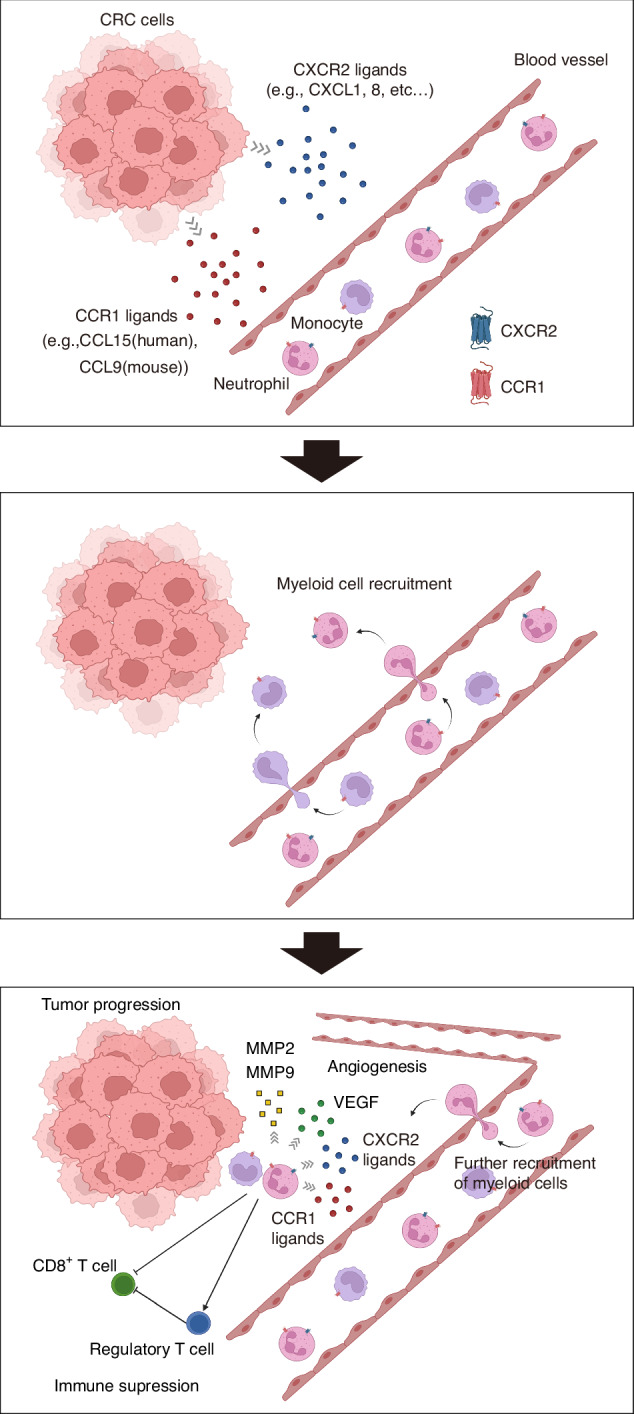
Schematic representation of synergistic effects of CCR1 and CXCR2 in CRC progression. In the TME of CRC, both CCR1 ligands (e.g., human CCL15 and mouse CCL9) and CXCR2 ligands (e.g., CXCL1 and human CXCL8) secreted from CRC cells recruits CCR1^+^ and/or CXCR2^+^ neutrophils from blood vessels. Thereafter, these recruited neutrophils promote tumor progression through immunosuppressive function (by decreasing CD8^+^ T cells and increasing regulatory T cell) and tissue-destructive and angiogenic function (by producing MMP2, MMP9 and VEGF). Created with BioRender.com.

Overall, our study provides a crucial foundation for the potential application of the dual blockade of CCR1 and CXCR2 in CRC therapy. The discovery of the synergistic effects of CCR1 and CXCR2 in myeloid cell recruitment (Fig. 8), as demonstrated by our double-knockout mice model for CCR1 and CXCR2, led us to the intriguing prospect of combining KM5908 and a CXCR2 inhibitor for CRC therapy. Although we recognize that there are potential challenges in applying these findings to human CRC patients (e.g., differences in the immune system between humans and mice, variations in CCR1 and CXCR2 expression levels among CRC patients, and immune-related adverse effects), the significant suppression of tumor growth and metastasis in a double-knockout mice model for CCR1 and CXCR2 suggests a promising direction for future research.

### Supplementary information

Supplemantary Figures and legends

Supplementary Tables

## Data Availability

The data and materials analyzed in the current study are available from the corresponding authors upon reasonable request.
