# Supplementary material for: Synergistic antitumor activity by dual blockade of CCR1 and CXCR2 expressed on myeloid cells within the tumor microenvironment
Source: Br J Cancer. 2024 May 15;131(1):63–76. doi: 10.1038/s41416-024-02710-x (PMC11231281; doi:10.1038/s41416-024-02710-x)
Supplement: Supplementary file 1 — Supplemantary Figures and legends [file 41416_2024_2710_MOESM1_ESM.pdf]

Supplementary Fig.1

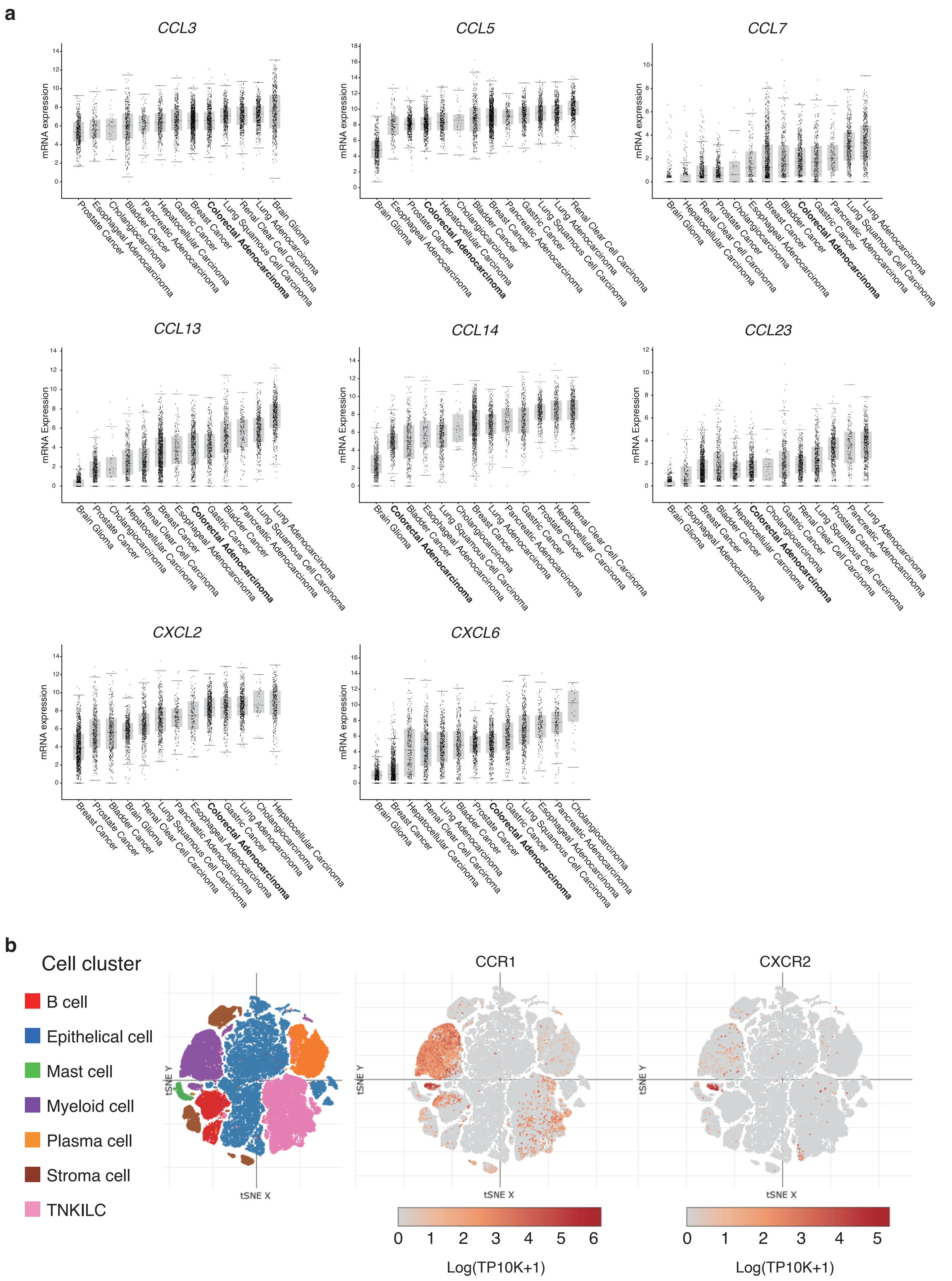

Supplementary Fig.2

a

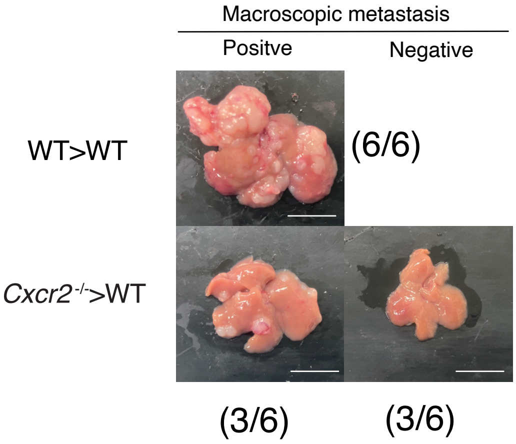

b

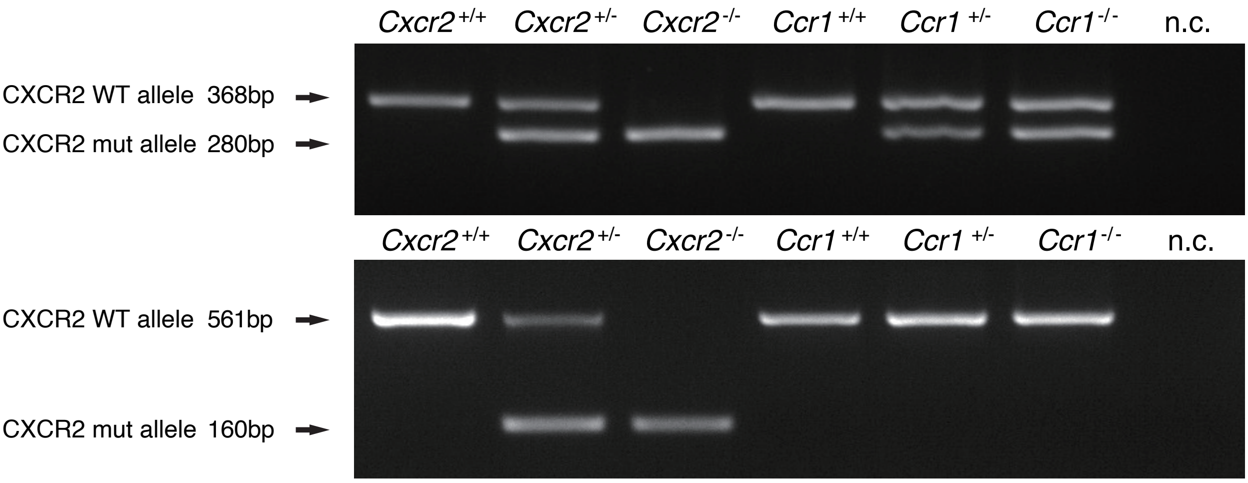

Supplementary Fig.3

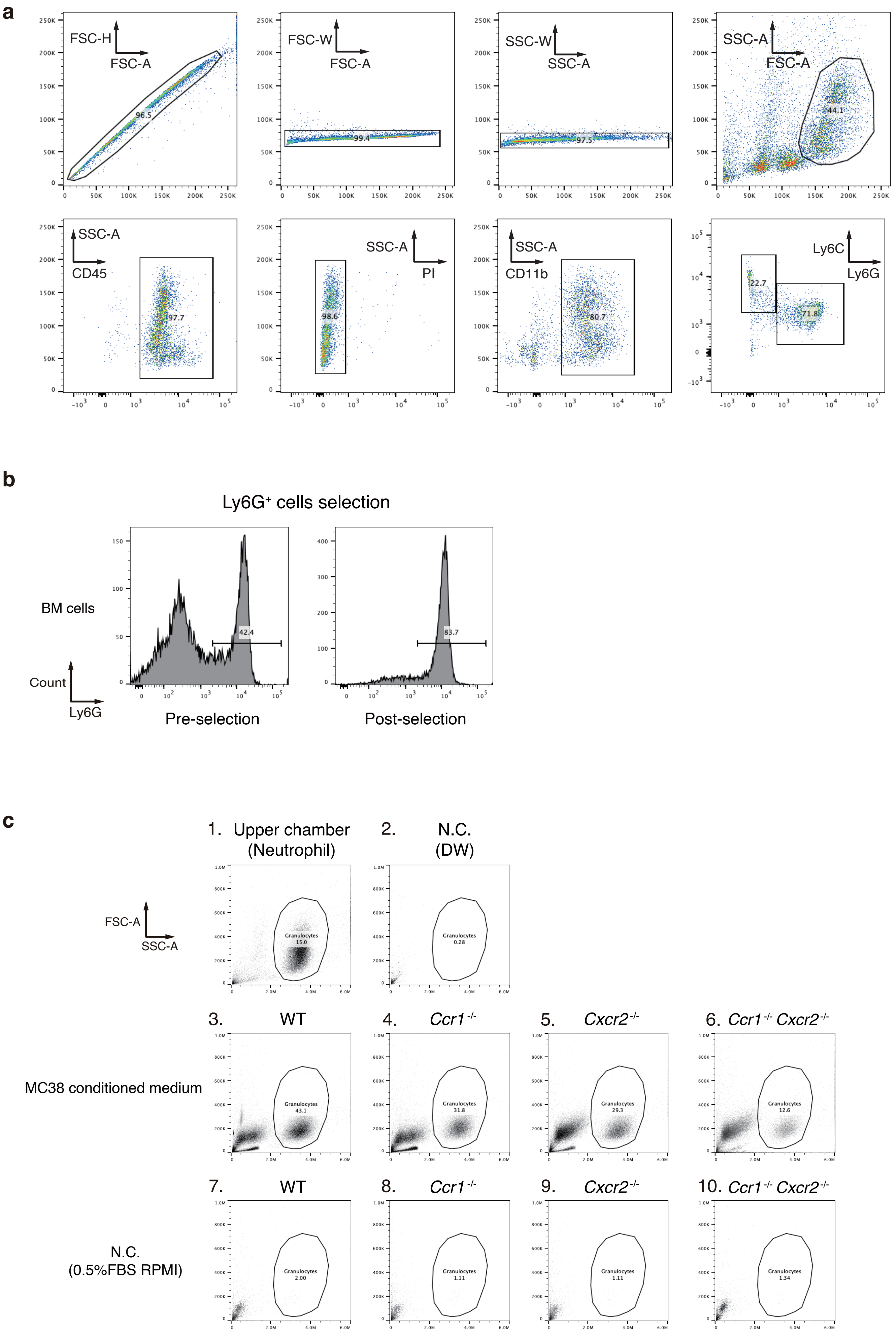

Supplementary Fig.4

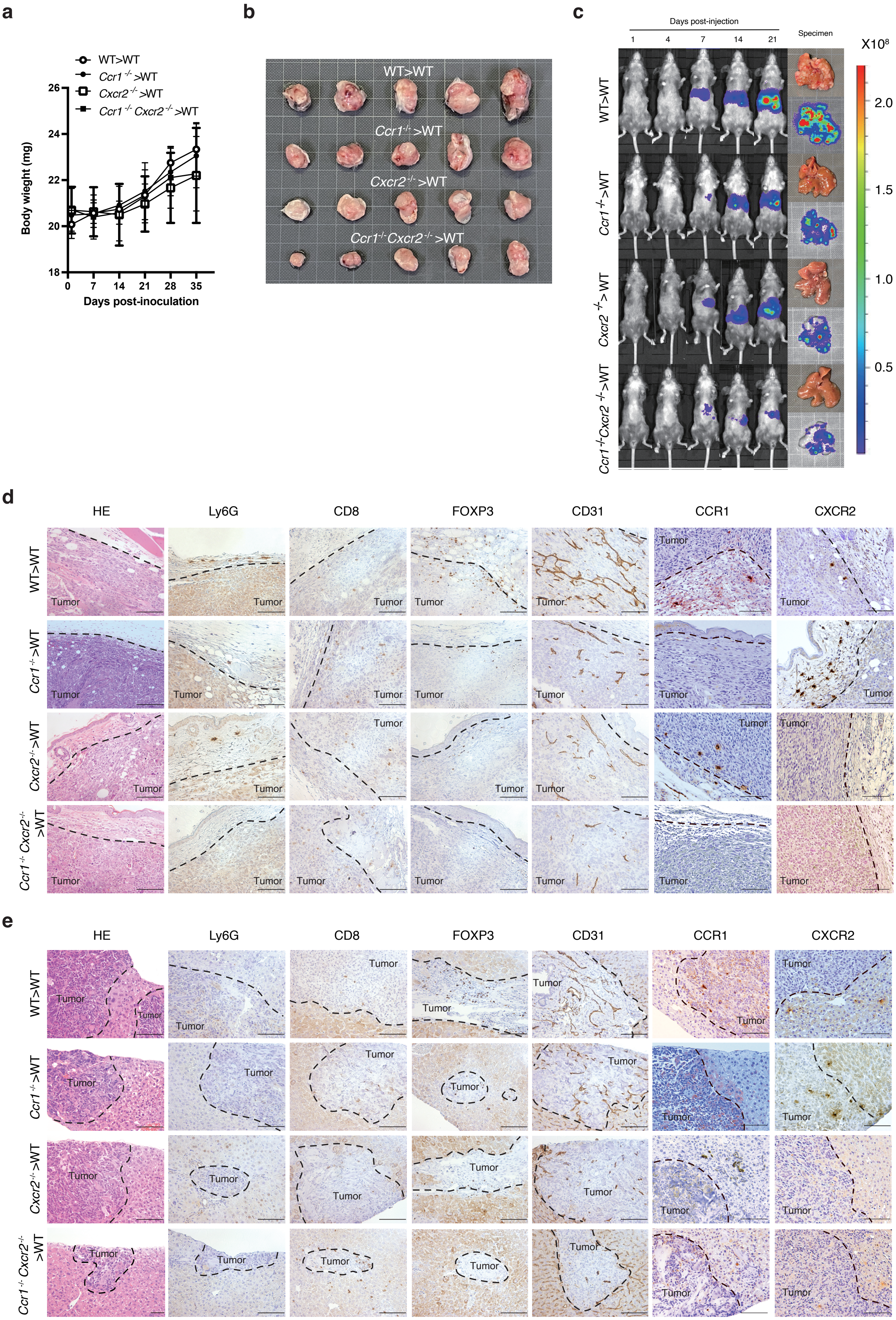

Supplementary Fig.5

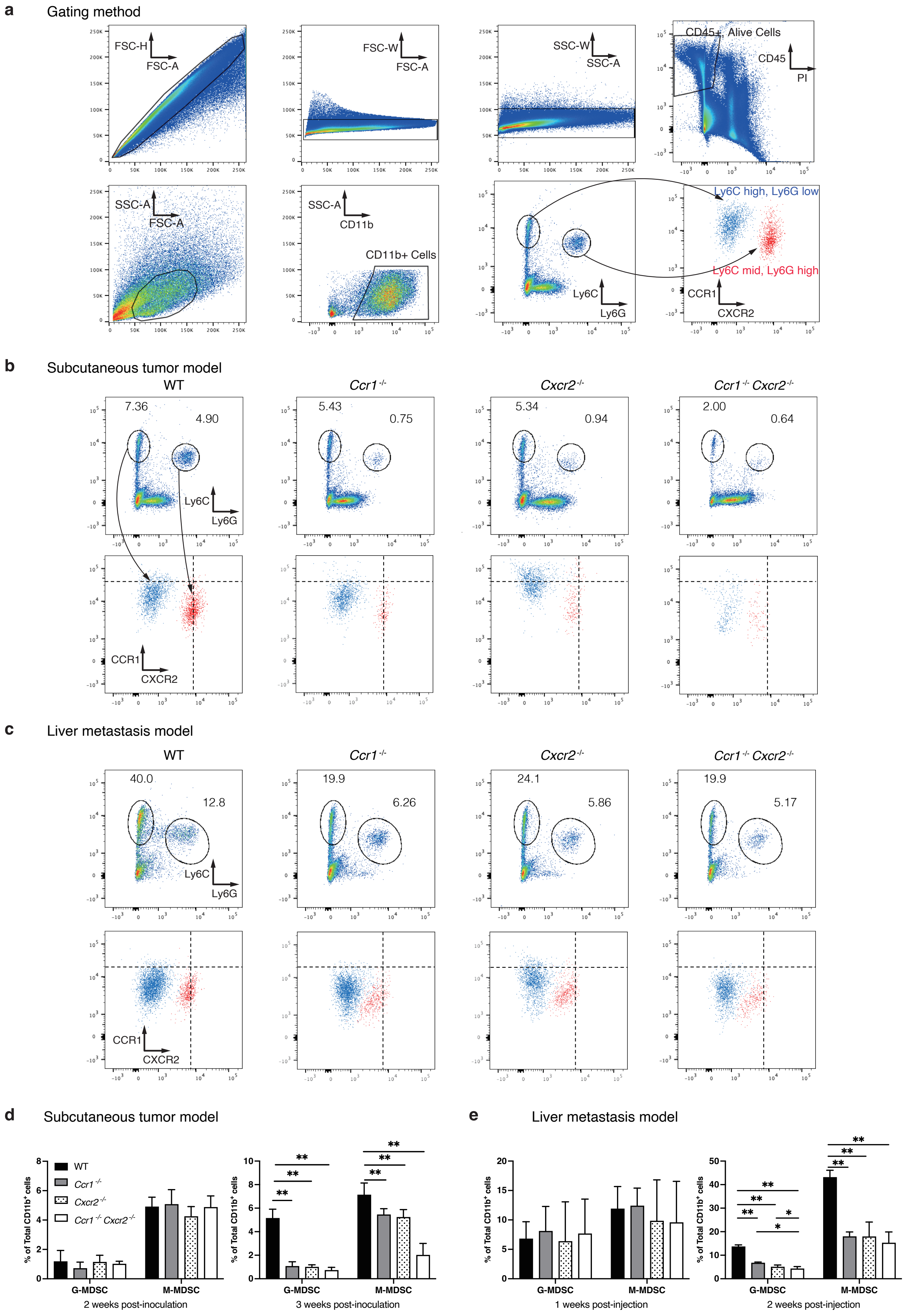

Supplementary Fig.6

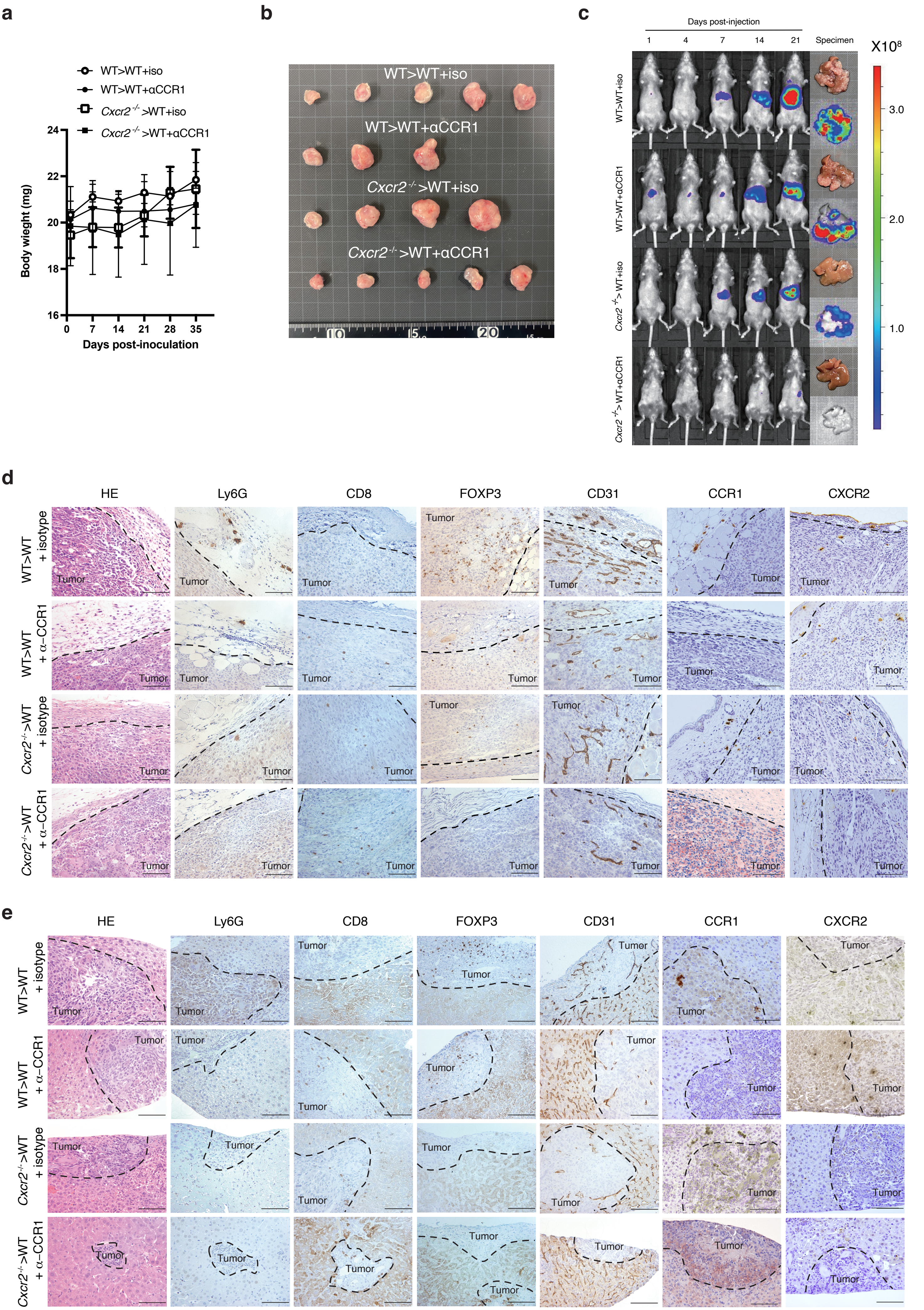

Supplementary Figure Legend

Supplementary Figure 1

a, Expression of CXCR2 ligands (CXCL2 and CXCL6) and CCR1 ligands (CCL3, CCL5, CCL7, CCL13, CCL14, and CCL23) in various types of cancer from TCGA database.

b, Expression profiles of CCR1 and CXCR2 across various cell clusters utilizing the Human Colon Cancer Atlas (c295) available at the Single Cell Portal. The analysis shows that, within the tumor microenvironment, CCR1 is expressed across the entirety of myeloid cells, while CXCR2 expression is observed in a subset of these cells.

Supplementary Figure 2

a, Representative macroscopic views of the livers dissected from WT>WT mice and *Cxcr2*$^{-/-}$>WT mice on day 21 post-injection. Note that all WT>WT mice (6 of 6) developed macroscopic foci, whereas metastases were observed only in half of *Cxcr2*$^{-/-}$>WT mice (3 of 6) ($P < 0.05$).

b, The primer set for CXCR2 mutated alleles (upper panel) provided by Jackson inadvertently detected the CCR1 mutated allele, primarily because both targeted the neomycin cassette. We thus designed a new CXCR2 primer set (lower panel) to specifically avoid incorrect detection of the CCR1 mutated allele, essential for the construction of the *Ccr1*$^{-/-}$*Cxcr2*$^{-/-}$ double-knockout mice.

Supplementary Figure 3

a, Flow cytometry gating strategy. First, single cells were selected. Next, neutrophils and monocytes were gated based on forward scatter (FSC-A) and side scatter (SSC-A) properties. Within this population, live cells were further gated for activation markers. Specifically, gates were set based on the expression levels of CD45, CD11b, Ly6G, Ly6C, CXCR2, and CCR1.

b, BM cells (left) were extracted, and neutrophils (right) were isolated using a positive selection methodology of Ly6G isolation.

c, Migration assay of neutrophils. The figure presents FACS plots, generated using FSC-A and SSC-A parameters, illustrating the varied migratory responses of neutrophils under different conditions and genetic backgrounds.

Upper lanes: Initial neutrophil population in the upper chamber and the negative control (N.C.) with distilled water (DW) respectively.

Middle lanes: Responses of neutrophils from WT, *Ccr1*$^{-/-}$, *Cxcr2*$^{-/-}$, and *Ccr1*$^{-/-}$ *Cxcr2*$^{-/-}$ mice toward MC38 conditioned medium.
Bottom lanes: Responses of neutrophils from WT, *Ccr1*$^{-/-}$, *Cxcr2*$^{-/-}$, and *Ccr1*$^{-/-}$ *Cxcr2*$^{-/-}$ mice toward N.C. medium (0.5% FBS in RPMI).

Supplementary Figure 4
a, Mouse body weight over time after tumor inoculation.
b, Images of transplanted MC38 tumors in WT>WT mice, *Ccr1*$^{-/-}$>WT mice, *Cxcr2*$^{-/-}$>WT mice and *Ccr1*$^{-/-}$*Cxcr2*$^{-/-}$>WT mice.
c, Representative *in vivo* bioluminescence images of MC38-luc liver metastases.
d and e, H&E and immunohistological staining for Ly6G^{+} neutrophils, CD8^{+} T cells, FOXP3^{+} Treg cells, CD31^{+} endothelial cells, CCR1^{+} cells and CXCR2^{+} cells around transplanted MC38 tumors (d) and MC38 liver metastases (e). Scale bar, 100 mm.

Supplementary Figure 5
a, Flow cytometry gating strategy of MC38 subcutaneous tumors in WT mice. The process begins with the selection of single cells. Next, cells are stained with CD45 and PI to gate the viable hematopoietic cells (CD45^{+}, alive cells). Following this, cells are further selected based on forward scatter (FSC-A) and side scatter (SSC-A) properties, and then myeloid cells are gated using CD11b. Subsequently, cell populations of granulocytic MDSCs and monocytic MDSCs are identified by their expression of Ly6C and Ly6G. Within this population, the expression of CXCR2 and CCR1 is verified. Similar processing can be applied to liver metastases.
b, The distribution of granulocytic MDSCs and monocytic MDSCs within the representative MC38 subcutaneous tumors from WT, *Ccr1*$^{-/-}$, *Cxcr2*$^{-/-}$ and *Ccr1*$^{-/-}$ *Cxcr2*$^{-/-}$ mice (upper panels), and the expression of CXCR2 and CCR1 (lower panels).
c, The distribution of granulocytic MDSCs and monocytic MDSCs within the representative MC38 liver metastases from WT, *Ccr1*$^{-/-}$, *Cxcr2*$^{-/-}$ and *Ccr1*$^{-/-}$ *Cxcr2*$^{-/-}$ mice (upper panels), and the expression of CXCR2 and CCR1 (lower panels).

d, Proportion of granulocytic MDSCs and monocytic MDSCs within the subcutaneous tumors at 2 and 3 weeks post-inoculation in each of the four groups (WT, *Ccr1*$^{-/-}$, *Cxcr2*$^{-/-}$ and *Ccr1*$^{-/-}$*Cxcr2*$^{-/-}$ mice).
e, Proportion of granulocytic MDSCs and monocytic MDSCs within the liver metastases at 1 and 2 weeks post-injection in each of the four groups (WT, *Ccr1*$^{-/-}$, *Cxcr2*$^{-/-}$ and *Ccr1*$^{-/-}$*Cxcr2*$^{-/-}$ mice).

Supplementary Figure 6
a, Mouse body weight over time after tumor inoculation.
b, Images of transplanted MC38 tumors from the four treatment groups: isotype-treated WT>WT mice, anti-CCR1-treated WT>WT mice, isotype-treated *Cxcr2*$^{-/-}$>WT mice and anti-CCR1-treated *Cxcr2*$^{-/-}$>WT mice.
c, Representative *in vivo* bioluminescence images of MC38-luc liver metastases in the four treatment groups: isotype-treated WT>WT mice, anti-CCR1-treated WT>WT mice, isotype-treated *Cxcr2*$^{-/-}$>WT mice and anti-CCR1-treated *Cxcr2*$^{-/-}$>WT mice.
d and e, H&E and immunohistological staining for Ly6G^{+} neutrophils, CD8^{+} T cells, FOXP3^{+} Treg cells, CD31^{+} endothelial cells, CCR1^{+} cells and CXCR2^{+} cells around transplanted MC38 tumors (d) and MC38 liver metastases (e). Scale bar, 100 mm.
