# Supplementary material for: Synergistic antitumor activity by dual blockade of CCR1 and CXCR2 expressed on myeloid cells within the tumor microenvironment
Source: Br J Cancer. 2024 May 15;131(1):63–76. doi: 10.1038/s41416-024-02710-x (PMC11231281; doi:10.1038/s41416-024-02710-x)
Supplement: Supplementary file 2 — Supplementary Tables [file 41416_2024_2710_MOESM2_ESM.docx]

**Supplementary Table S1.**

| mouse CCR1 forward | 5’-TCCTCAGCAAAGGATGGAGA-3’ |
| --- | --- |
| mouse CCR1 reverse | 5’-CAGACGCACGGCTTTGACCTTCTT-3’ |
| mouse CXCR2 WT-forward-1 | 5’-GGTCGTACTGCGTATCCTGCCTCA-3’ |
| mouse CXCR2WT-reverse-1 | 5’-TAGCCATGATCTTGAGAAGTCCAT-3’ |
| mouse CXCR2 Mut-forward-1 | 5’-CTTGGGTGGAGAGGCTATTC-3’ |
| mouse CXCR2 Mut-reverse-1 | 5’-AGGTGAGATGACAGGAGATC-3’ |
| mouse CXCR2 WT-forward-2 | 5’-GGGAGAGGGAAGGGAATAGG-3’ |
| mouse CXCR2WT-reverse-2 | 5’-GCAAGAATGTGGGAATACCAG-3’ |
| mouse CXCR2 Mut-forward-2 | 5’-GGCATTCTGCACGCTTCAAAAGCGC-3’ |
| mouse CXCR2 Mut-reverse-2 | 5’-GCCCAGTCATAGCCGAATAGCCTCTCC-3’ |

**Supplementary Table S2.**

| mouse/rat *Gapdh*-forward | 5’-TGTCCGTCGTGGATCTGAC-3’ |
| --- | --- |
| mouse/rat *Gapdh*-reverse | 5’-CCTGCTTCACCACCTTCTTG-3’ |
| mouse *Cxcl1*-forward | 5’-ATCCAGAGCTTGAAGGTGTTG-3’ |
| mouse *Cxcl1*-reverse | 5’-GTCTGTCTTCTTTCTCCGTTACTT-3’ |
| rat *Cxcl1*-forward | 5’-ATCCAGAGTTTGAAGGTGATG-3’ |
| rat *Cxcl1*-reverse | 5’-ATCTATCTTCTTTCTCCATTACTT-3’ |
| mouse *Cxcl2*-forward | 5’-ATGCCTGAAGACCCTGCCAAG-3’ |
| mouse/rat *Cxcl2*-reverse | 5’-GGTCAGTTAGCCTTGCCTTTG-3’ |
| rat *Cxcl2*-forward | 5’-ATGCCTGACGACCCTCCCAAG-3’ |
| mouse/rat *Cxcl3*-forward | 5’-CATCCAGAGCTTGACGGTGAC-3’ |
| mouse *Cxcl3*-reverse | 5’-CTTGCCGCTCTTCAGTATCTTCTT-3’ |
| rat *Cxcl3*-reverse | 5’-CTTGTCACTCTTCAGTAACTTCTG-3’ |
| mouse *Cxcl5*-forward | 5’-GCATTTCTGTTGCTGTTCACGCT-3’ |
| mouse *Cxcl5*-reverse | 5’-GGTTAAGCAAACACAACGCAGCT-3’ |
| rat *Cxcl5*-forward | 5’-GCATTTCTGCTGCTGTTCACACT-3’ |
| rat *Cxcl5*-reverse | 5’-GGTTAAGCAAACACAGCGTAGCT-3’ |
| mouse *Cxcl7*-forward | 5’-GGAAAATCTGATGGCATGGAC-3’ |
| mouse *Cxcl7*-reverse | 5’-CAGGCACGTTTTTTGTCCATTCT-3’ |
| rat *Cxcl7*-forward | 5’-ATGGGCTTCAGACTCAGACCTA-3’ |
| rat *Cxcl7*-reverse | 5’-AACACATTCACACGGGAGATAG-3’ |
| mouse *Ccl3*-forward | 5’-ACTGCCTGCTGCTTCTCCTACA-3’ |
| mouse *Ccl3*-reverse | 5’-ATGACACCTGGCTGGGAGCAAA-3’ |
| rat *Ccl3*-forward | 5’-ACACCCCGACTGCCTGCTGCTT-3’ |
| rat *Ccl3*-reverse | 5’-CTGCCGGTTTCTCTTGGTCAGG-3’ |
| mouse *Ccl4*-forward | 5’-ACCCTCCCACTTCCTGCTGTTT-3’ |
| mouse *Ccl4*-reverse | 5’-CTGTCTGCCTCTTTTGGTCAGG-3’ |
| rat *Ccl4*-forward | 5’-ACCCTCCCACTTCCTGCTGCTT-3’ |
| rat *Ccl4*-reverse | 5’-TTGCCTGCCTTTTTTGGTCAGA-3’ |
| mouse *Ccl5*-forward | 5’-GCTGCCCTCACCATCATCCTCACT-3’ |
| mouse *Ccl5*-reverse | 5’-GGCACACACTTGGCGGTTCCTTC-3’ |
| rat *Ccl5*-forward | 5’-ACCACTCCCTGCTGCTTT-3’ |
| rat *Ccl5*-reverse | 5’-ACACTTGGCGGTTCCTTCG-3’ |
| mouse *Ccl6*-forward | 5’-CACCAGTGGTGGGTGCATCAAG-3’ |
| mouse *Ccl6*-reverse | 5’-GTGCTTAGGCACCTCTGAACTC-3’ |
| rat *Ccl6*-forward | 5’-AACCAGTGGTGGGTGCACCAAG-3’ |
| rat *Ccl6*-reverse | 5’-GTGCTTATGCACGTCTGAACTC-3’ |
| mouse/rat *Ccl7*-forward | 5’-CAGAAGGATCACCAGTAGTCGG-3’ |
| mouse/rat *Ccl7*-reverse | 5’-ATAGCCTCCTCGACCCACTTCT-3’ |
| mouse *Ccl9*-forward | 5’-ATGAAGCCTTTTCATACTGCCCTC-3’ |
| mouse *Ccl9*-reverse | 5’-TTATTGTTTGTAGGTCCGTGGTTG-3’ |
| rat *Ccl9*-forward | 5’-ATGAAGCCCTTTCATACTGCCCTC-3’ |
| rat *Ccl9*-reverse | 5’-TTATTGTTTGTAGATCTGTGGTTG-3’ |
| mouse Ccr1-foward | 5’-AGCCTGAAGCAGTGGAAGAG-3’ |
| mouse Ccr1-reverse | 5’-CAGACGCACGGCTTTGACCTTCTT-3’ |
| mouse Cxcr2-foward | 5’-GAAATTTCGCCATGGACTTCTC-3’ |
| mouse Cxcr2-reverse | 5’-ACGAGCTAACAAAAGAAGGCCTT-3’ |

**Supplementary Table S3.**

List of Antibodies used for FACS.

| **Antibody** | **Host** | **Conjugate** | **Clone** | **Company** |
| --- | --- | --- | --- | --- |
| anti-CD45 | rat | Apc-Cy7 | 30-F11 | BioLegend |
| anti-CD11b | rat | PE-Cy7 | M1/70 | BD Pharmingen |
| anti-Ly6G | rat | FITC | 1A8 | BD Pharmingen |
| anti-Ly6C | rat | BV510 | HK1.4 | BioLegend |
| anti-CXCR2 | rat | PE | SA044G4 | BioLegend |
| Anti-CCR1 | mouse | Alexa Fluor 647 |  | Kyowa Kirin Co., Ltd. |

**Supplementary Table S4.**

List of Antibodies used for IHC.

| **Primary Antibody** | **Host** | **Company** | **Dilution (IHC)** |
| --- | --- | --- | --- |
| anti-mLy6G | rat | BioXcell | 1:1000 |
| anti-mCD8 | rat | eBiosciene | 1:100 |
| anti-mFOXP3 | rabbit | Cell Signaling | 1:100 |
| anti-mCD31 | rat | Cell Signaling | 1:100 |
| anti-mCCR1 | rabbit | made in our lab^4,7^ | 1:500 |
| anti-mCXCR2 | rat | R&D Systems | 1:100 |
| **Secondary Antibody** | **Host** | **Company** | **Dilution (IF)** |
| anti-rabbit | goat | Vector Laboratories | 1:200 |
| anti-rat | rabbit | Dako | 1:200 |

**Supplementary Table S5.**

List of Antibodies used for IF.

| **Primary Antibody** | **Host** | **Company** | **Dilution (IF)** |
| --- | --- | --- | --- |
| anti-mCCR1 | rabbit | made in our lab^4,7^ | 1:500 |
| anti-mCXCR2 | rat | R&D Systems | 1:100 |
| anti-mMMP2 | goat | R&D Systems | 1:20 |
| anti-mMMP9 | goat | R&D Systems | 1:20 |
| anti-mVEGF | goat | R&D Systems | 1:200 |
| **Secondary Antibody** | **Host** | **Company** | **Dilution (IF)** |
| anti-rabbit | goat | Invitrogen | 1:500 |
| anti-rat | goat | Invitrogen | 1:500 |
| anti-goat | rabbit | Invitrogen | 1:500 |
